# C/EBPα and GATA-2 Mutations Induce Bilineage Acute Erythroid Leukemia through Transformation of a Neomorphic Neutrophil-Erythroid Progenitor

**DOI:** 10.1016/j.ccell.2020.03.022

**Published:** 2020-05-11

**Authors:** Cristina Di Genua, Simona Valletta, Mario Buono, Bilyana Stoilova, Connor Sweeney, Alba Rodriguez-Meira, Amit Grover, Roy Drissen, Yiran Meng, Ryan Beveridge, Zahra Aboukhalil, Dimitris Karamitros, Mirjam E. Belderbos, Leonid Bystrykh, Supat Thongjuea, Paresh Vyas, Claus Nerlov

**Affiliations:** 1 MRC Molecular Haematology Unit, MRC Weatherall Institute of Molecular Medicine, University of Oxford, John Radcliffe Hospital, Headington, Oxford OX3 9DS, UK; 2 Princess Máxima Center for Pediatric Oncology, 3584 CS Utrecht, the Netherlands; 3 European Research Institute for the Biology of Ageing, University Medical Center Groningen, 9713 AV Groningen, the Netherlands; 4 MRC WIMM Centre for Computational Biology, MRC Weatherall Institute of Molecular Medicine, University of Oxford, Oxford OX3 9DS, UK; 5 NIHR Oxford Biomedical Research Center, John Radcliffe Hospital, University of Oxford, Oxford OX3 9DU, UK

**Keywords:** acute myeloid leukemia, acute erythroid leukemia, leukemia-initiating cell, lineage priming, chromatin accessibility, oncogene co-operation, *CEBPA*, *GATA2*

## Abstract

Acute erythroid leukemia (AEL) commonly involves both myeloid and erythroid lineage transformation. However, the mutations that cause AEL and the cell(s) that sustain the bilineage leukemia phenotype remain unknown. We here show that combined biallelic *Cebpa* and *Gata2* zinc finger-1 (ZnF1) mutations cooperatively induce bilineage AEL, and that the major leukemia-initiating cell (LIC) population has a neutrophil-monocyte progenitor (NMP) phenotype. In pre-leukemic NMPs *Cebpa* and *Gata2* mutations synergize by increasing erythroid transcription factor (TF) expression and erythroid TF chromatin access, respectively, thereby installing ectopic erythroid potential. This erythroid-permissive chromatin conformation is retained in bilineage LICs. These results demonstrate that synergistic transcriptional and epigenetic reprogramming by leukemia-initiating mutations can generate neomorphic pre-leukemic progenitors, defining the lineage identity of the resulting leukemia.

## Highlights

• Biallelic C/EBPα and GATA-2 ZnF1 mutations synergize during leukemogenesis
• GATA-2 ZnF1 mutation generates an erythroid-permissive chromatin state
• C/EBPα and GATA-2 mutant NMPs show ectopic erythroid lineage potential
• Transformed leukemic NMPs are bipotent neutrophil-erythroid leukemia-initiating cells

## Graphical Abstract

**Figure:**
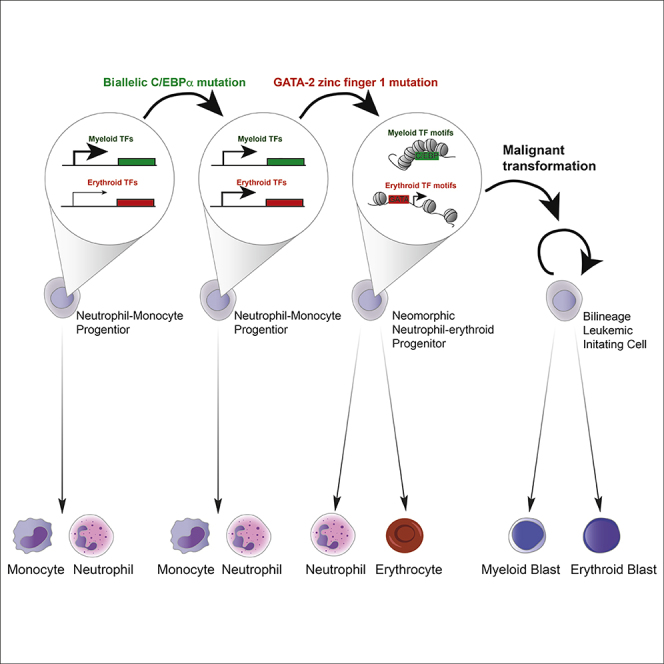

## Teaser

By combining biallelic C/EBPα and GATA-2 ZnF1 mutations, Di Genua et al. generate a mouse model of bilineage acute erythroid leukemia and identify a neutrophil-monocyte progenitor (NMP) that undergoes transcriptional and epigenetic reprogramming to express erythroid genes as the major leukemia-initiating cell.

## Significance

**We here show that, together, *Cebpa* and *Gata2* mutations can cause bilineage AEL in mice, and that the resulting leukemia is cellularly and molecularly analogous to human AEL. We also show AEL is maintained by self-renewing leukemia-propagating cells that remain bipotent at the single-cell level, and thus generate a bilineage differentiation hierarchy. In addition, we identify a mechanism whereby transcriptional and epigenetic changes, induced by *Cebpa* and *Gata2* mutation, respectively, synergize to define the lineage identity of the resulting leukemia. Together, these findings generate a cellular and molecular framework for the etiology of, and provide a pre-clinical model for, bilineage AEL, and underscore the importance of studying the pre-leukemic state for understanding oncogene collaboration during leukemogenesis.**

## Introduction

Acute myeloid leukemia (AML) arises through the sequential acquisition of somatic mutations, most initially occurring in the self-renewing hematopoietic stem cell (HSC) compartment, and subsequently in the progenitor cells that undergo transformation (Jan et al., 2012). This leads to the pathological accumulation of immature cells, arrested in differentiation, that ultimately displace normal hematopoiesis. AML is both genetically and morphologically heterogeneous. More than 20 genes are commonly mutated in AML, with on average 5 such acquired mutations observed in each tumor (Cancer Genome Atlas Research, 2013), giving rise to monocytic, neutrophil, erythroid, and megakaryocytic (Bennett et al., 1976), and more rarely basophil/mast cell and eosinophil leukemia (Lichtman and Segel, 2005).

Gene expression profiling identified 16 transcriptional AML subtypes, many correlated with specific driver mutations, including *FLT3*, *RUNX1*, *CEBPA*, and *MLL1* mutations (Valk et al., 2004). Furthermore, 11 distinct mutational patterns were observed (Papaemmanuil et al., 2016), including association of *NPM1* mutation with mutations involved in DNA methylation, and *RUNX1* and *CBFB* translocations with *KIT* and *NRAS* mutation. In addition, specific association of *CEBPA* mutation with *GATA2* zinc finger-1 (ZnF1) mutation, distinct from the *GATA2* ZnF2 mutations associated with MonoMAC syndrome (Hsu et al., 2011), was observed (Metzeler et al., 2016, Papaemmanuil et al., 2016), whereas other common mutations (*FLT3-ITD*, *NPM1*, *MLL*, *RUNX1*, and *IDH1/2*) were negatively correlated to biallelic *CEBPA* mutation (Fasan et al., 2014). Targeted sequencing confirmed the prevalence of *GATA2* ZnF1 mutations in *CEBPA* mutant AML, with additional common mutations observed only in a minority (6/35) of patients (Fasan et al., 2013, Greif et al., 2012, Ping et al., 2017). Interestingly, while the majority of patients carrying *GATA2* mutations were of a granulocytic (M1 or M2) subtype, mutations were also observed in acute erythroid leukemia (AEL) (AML M6 subtype) (Fasan et al., 2013). In AEL there was a specific and statistically significant association of biallelic *CEBPA* mutation to *GATA2* ZnF1 mutation, as well as a higher incidence of *GATA2* ZnF1 mutation compared with non-AEL AML (Ping et al., 2017).

This indicated that combined *CEBPA* and *GATA2* mutations contribute to the etiology of both myeloblastic and erythroid acute leukemias. AEL in its most common form is bilineage, characterized by the presence of both myeloblasts (MBs) and erythroblasts blocked in their differentiation (Arber et al., 2008, Zuo et al., 2010). However, while several studies have identified recurrent mutations in AEL tumors (Cervera et al., 2016, Ping et al., 2017, Santos et al., 2009), and erythroid lineage transformation has been successfully modeled (Iacobucci et al., 2019, Thoene et al., 2019), so far no mutations have been identified as causative of bilineage AEL. M1 and M2 AML subtypes, which are also those principally observed to contain biallelic *CEBPA* mutations (Valk et al., 2004), are generated by transformation of the neutrophil granulocyte lineage. Murine studies have shown that neutrophil differentiation progresses via progenitors committed to a neutrophil/monocyte fate (neutrophil-monocyte progenitors or NMPs), where *Gata2* expression is low or absent (Drissen et al., 2016). Conversely, erythroid lineage progenitors express high levels of *Gata2*, but lack *Cebpa* expression (Pronk et al., 2007). This raises the question of how, and in which cell type, synergy between *CEBPA* and *GATA2* mutations is achieved, and in particular whether the bilineage leukemia phenotype is maintained by a single bipotent, or by two distinct lineage-restricted, leukemia-propagating cell populations.

Two types of *CEBPA* mutations are observed in AML: N-terminal mutations leading to selective loss of the C/EBPα 42 kDa isoform (p42) while preserving translation of the 30-kDa isoform (p30), and C-terminal mutations that disable DNA binding of both C/EBPα p42 and p30, while preserving the leucine zipper dimerization domain. Both types of mutations impair the ability of C/EBPα to block cell-cycle progression via E2F repression (Lopez et al., 2009). Patients with biallelic *CEBPA* mutation most commonly carry one mutation of each type (Nerlov, 2004, Wouters et al., 2009). We have previously modeled biallelic *CEBPA* mutant AML in the mouse and observed that the combination of N- and C-terminal C/EBPα mutation is optimal for leukemogenesis (Bereshchenko et al., 2009), consistent with the clinically observed mutation pattern. This combination of *Cebpa* mutations both decreases HSC quiescence, leading to expansion of pre-malignant HSCs, and allows myeloid lineage commitment (Bereshchenko et al., 2009). Myeloid lineage commitment is important for leukemogenesis, as *Cebpa* mutant leukemias are propagated by committed myeloid progenitors (Bereshchenko et al., 2009, Kirstetter et al., 2008) whose self-renewal is dramatically increased by loss of C/EBPα-mediated E2F repression (Porse et al., 2005), and requires the p30 isoform, which retains the SWI/SNF binding domain critical for activation of C/EBP-dependent myeloid lineage genes (Pedersen et al., 2001). Complete loss of C/EBPα consequently does not induce AML due to lack of granulocyte-monocyte progenitor formation (Zhang et al., 2004).

In contrast little is known about the role of *GATA2* ZnF1 mutations in myeloid leukemogenesis. GATA-2 ZnF1 is known to interact with other transcription factors (TFs), including FOG-1 (Chang et al., 2002) and LMO2 (Osada et al., 1995). However, the ZnF1 residues mutated in AML (Fasan et al., 2013, Greif et al., 2012, Papaemmanuil et al., 2016, Ping et al., 2017) do not correspond to those that interact with FOG-1 or LMO2 (Wilkinson-White et al., 2011). The molecular and cellular consequences of *GATA2* ZnF1 mutations therefore still need to be identified, and so far no genetic model of this mutation has been generated.

To understand the role of *GATA2* ZnF1 mutations in myeloid leukemogenesis, and to model human bilineage AEL, we therefore generated a murine genetic model of combined biallelic *CEBPA* and *GATA2* ZnF1 mutation.

## Results

### Generation of an Accurate Model of Combined *CEBPA* and *GATA2* Mutant AML

To model combined *CEBPA* and *GATA2* ZnF1 mutations we generated a murine germ line knock-in allele of the human *GATA2* G320D mutation (henceforth *Gata2*^D^ allele) that was observed in conjunction with biallelic *CEBPA* mutation in multiple studies (Fasan et al., 2013, Greif et al., 2012, Papaemmanuil et al., 2016, Ping et al., 2017) (Figure S1A). *GATA2* ZnF1 mutations are heterozygous (Greif et al., 2012), and consistent with this we observed that homozygosity, but not heterozygosity, for the *Gata2*^D^ allele led to loss of HSC self-renewal (Figures S1B–S1E). We therefore combined a single *Gata2*^D^ allele with the previously described N- and C-terminal *Cebpa* knock-in mutations (*Cebpa*^L^ [Kirstetter et al., 2008] and *Cebpa*^K^ alleles [Bereshchenko et al., 2009], respectively) to generate triple knock-in mice carrying biallelic *Cebpa* and heterozygous *Gata2* ZnF1 mutation (*Cebpa*^K/L^; *Gata2*^D/+^ or KLG genotype), as well as *Cebpa*^K/L^ (KL genotype) and *Gata2*^D/+^ (G genotype) mice. Because of the perinatal lethality of the *Cebpa*^K/L^ mutation we generated embryonic day 14.5 fetal liver (FL) cells with these genotypes, and wild-type (WT) control FLs (CD45.2 allotype). These were competitively transplanted into lethally irradiated recipients (CD45.1/2 allotype) using CD45.1/2 WT competitor, as described previously (Bereshchenko et al., 2009) (Figure S2A). Where indicated the CD45.1/2 allotype was combined with the *Gata1*-EGFP transgene that efficiently labels platelets and erythroid cells (Carrelha et al., 2018, Drissen et al., 2016), allowing experimental, CD45.2-derived erythroid lineage cells (EGFP^–^) to be distinguished from competitor- and recipient-derived erythroid cells (EGFP^+^), and therefore the development of erythroid lineage phenotypes in *Cebpa* and *Gata2* mutant cells to be observed. Mice transplanted with FL cells of the four genotypes were monitored by periodic peripheral blood (PB) analysis (Figures S2B–S2E). This analysis showed comparable overall reconstitution of PB leukocytes by all four genotypes (Figure S2F). However, mice transplanted with KLG FL cells (henceforth KLG mice) showed increased myeloid contribution after 20 weeks, with no significant differences in lymphoid cell contribution (Figure S2F). In addition, both KL and KLG mice showed more rapid reconstitution of erythrocytes, but not of platelets (Figure S2G).

### Biallelic *Cebpa* and *Gata2* ZnF1 Mutations Synergistically Induce Bilineage AEL

**Figure 1:**
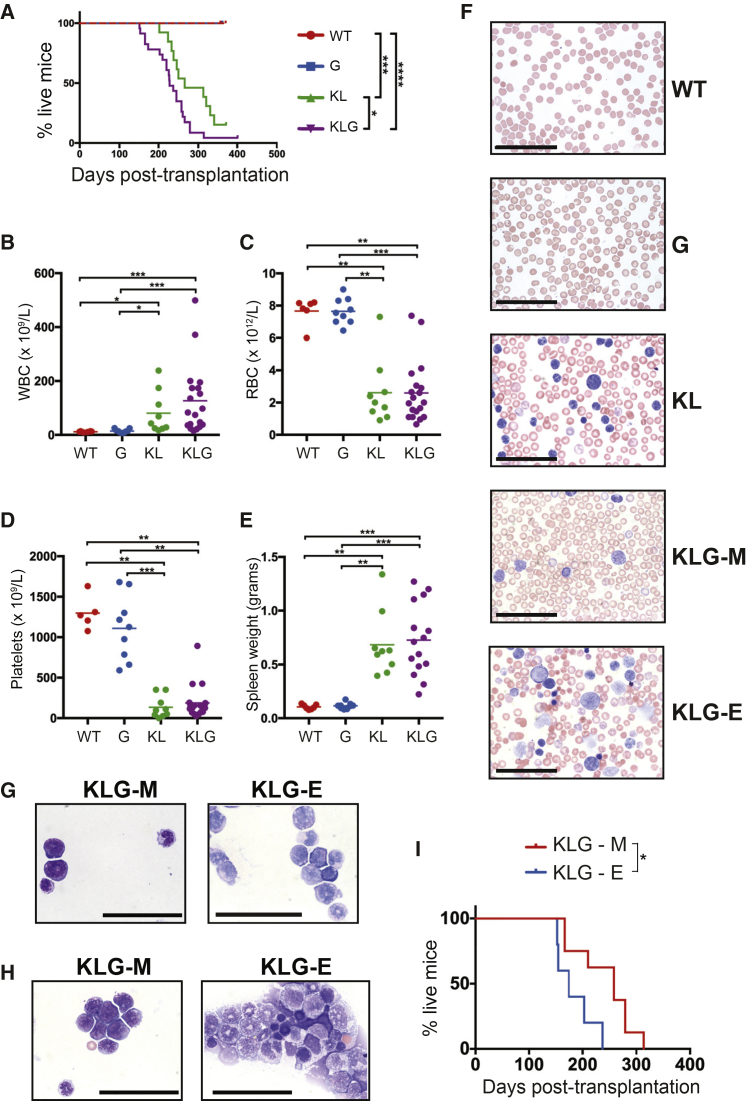
Biallelic *Cebpa* and *Gata2* ZnF1 Mutations Synergistically Induce Erythroid Leukemia (A) Event-free survival. Differences in survival were analyzed using a Mantel-Cox log-rank test. (B) White blood cell count in mice from (A). Parameters were measured during terminal analysis. Leukemic mice were analyzed when moribund, non-leukemic mice at 52 weeks post-transplantation. WT, n = 7; G, n = 9; KL, n = 13; KLG, n = 23 in four independent experiments. The mean and significant differences between genotypes are indicated. (C) Red blood cell (RBC) count in mice from (A). (D) Platelet count in mice from (A). (E) Spleen weight in mice from (A). (F) Representative PB smears from mice in (A). (G) Representative BM cytospins from mice in (A). (H) Representative spleen cytospins from mice in (A). Blood smears and cytospins were stained with May-Grünwald and Giemsa. Analysis is representative of three replicates per genotype from a total of four independent experiments. (I) Event-free survival comparison of KLG-M (n = 8) and KLG-E (n = 5) mice performed as in (A). ^∗^p < 0.05, ^∗∗^p < 0.01, ^∗∗∗^p < 0.001, ^∗∗∗∗^p < 0.0001. (F–H) Scale bars, 50 μm. See also Figures S1 and S2 and Table S1.

Consistent with accelerated myeloid lineage output from transplanted KLG FL cells, KLG mice developed leukemia more rapidly (Figure 1A; average latency of 8 months) than KL mice (average latency of 10 months) (Table S1). No leukemia was observed in WT or G mice. Moribund mice were characterized by increased leukocyte count (Figure 1B), anemia (Figure 1C), thrombocytopenia (Figure 1D), and splenomegaly (Figure 1E), consistent with AML. Examination of blood smears from leukemic mice showed the presence of leukemic blasts. However, while KL blasts were consistently myeloid (Figure 1F), 5/13 of the examined leukemic KLG mice contained both myeloid and erythroid blast cells in PB (KLG-E mice), with the remaining mice showing only myeloid blast morphology (KLG-M mice). The same pattern was observed in bone marrow (BM) (Figure 1G) and spleen (Figure 1H). In addition, KLG-E mice showed prominent dyserythropoiesis (Figure 1F), a characteristic feature of AEL (Zuo et al., 2010). Comparison of survival of KLG-M and KLG-E mice showed that KLG-E leukemias developed faster than the purely myeloid KLG-M leukemias (Figure 1I).

**Figure 2:**
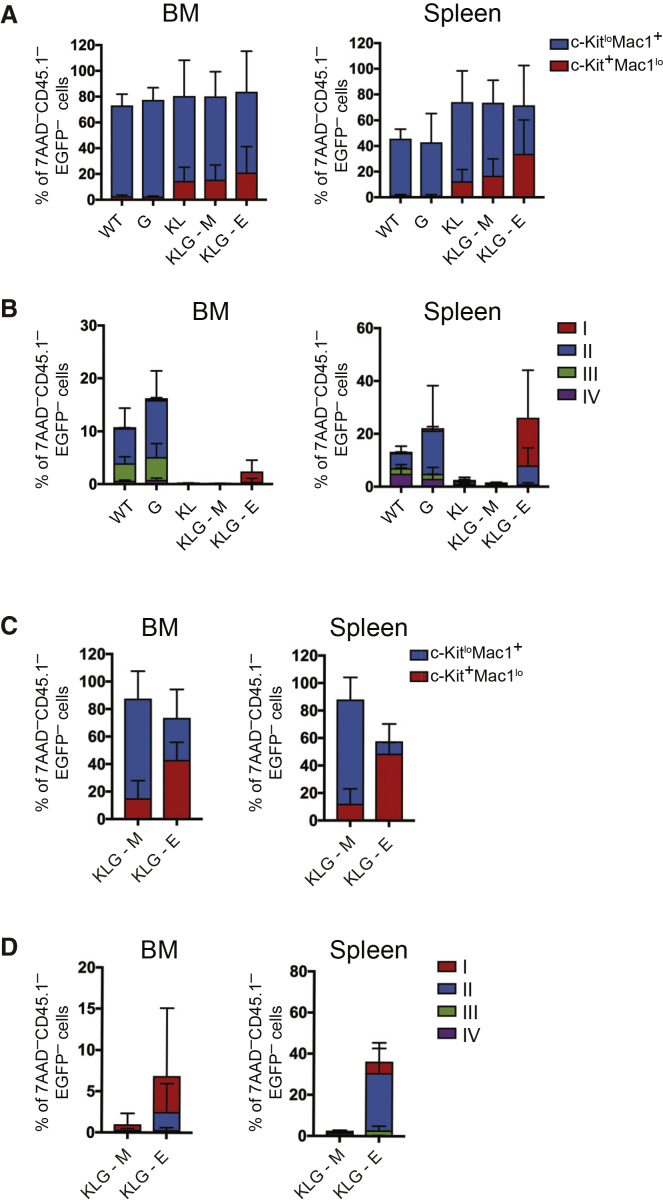
KLG-E Mice Contain Both Myeloblasts and Erythroblasts in the BM and Spleen (A) Histogram showing c-Kit^lo^Mac1^+^ and c-Kit^+^Mac1^lo^ cells as a percentage of 7AAD^–^CD45.1^–^EGFP^–^ cells in the BM (left panel) and spleen (right panel) in primary transplanted mice. WT, n = 7 (non-leukemic); G, n = 7 (non-leukemic); KL, n = 3 (all leukemic); KLG-M, n = 6 (all leukemic); KLG-E, n = 4 (all leukemic) from a total of three independent experiments. (B) Histogram showing stage I–IV erythroblast cells as a percentage of 7AAD^–^CD45.1^–^EGFP^–^ cells in the BM (left panel) and spleen (right panel) in primary transplanted mice from (A). (C) Histogram showing c-Kit^lo^Mac1^+^ and c-Kit^+^Mac1^lo^ cells as a percentage of 7AAD^–^CD45.1^–^EGFP^–^ cells in the BM (left panel) and spleen (right panel) of mice transplanted with KLG-M and KLG-E leukemias, as indicated. Cell numbers transplanted are shown in Table S2. Five mice were analyzed for each leukemia phenotype. (D) Histogram showing stage I–IV erythroblasts cells as a percentage of 7AAD^–^CD45.1^–^EGFP^–^cells in the BM (left panel) and spleen (right panel) of mice from (C). The results are presented as the mean ± SD. See also Figures S3 and S4 and Table S2.

Analysis by flow cytometry showed a significant expansion of mutant CD45.2 immature c-Kit^+^ Mac-1^lo^ myeloid cells in all leukemic mice in both BM and spleen (Figures 2A and S3A–S3C), with corresponding loss of Ter119^+^ stage II–IV erythroid progenitors (Figures 2B and S3A, S3D, and S3E). Importantly, in leukemic KLG-E mice, but not KL or KLG-M mice, expansion of immature CD45.1^–^EGFP^–^ (i.e., CD45.2 donor-derived) CD71^hi^Ter119^lo^ erythroblast (corresponding to erythroblast fraction I; Socolovsky et al., 2001) was observed in BM, and to an even greater extent in spleen (>20% erythroblasts; Figure 2B). These CD71^hi^Ter119^lo^ immature erythroblasts were c-Kit^+^ (Figures S4A–S4D) and accumulated in high numbers in the spleen (Figures S4C and S4D). Combined with the absence of EGFP^–^ stage III–IV erythroid progenitors this was consistent with the morphologically observed accumulation of immature, leukemic erythroid progenitors in KLG-E BM, spleen, and blood. Finally, transplantation of KLG-M leukemia cells into irradiated recipients generated a purely myeloid leukemia (Figures 2C, 2D, and S4E–S4H) within 8 weeks (Table S2) with remaining CD45.2-derived CD45.1^–^EGFP^–^ erythroid cells (most likely derived from residual pre-leukemic HSCs; Bereshchenko et al., 2009) showing a normal differentiation profile (Figure S4H), whereas mice transplanted with KLG-E leukemia cells developed leukemia faster, with an average latency of 5 weeks (Table S2), and accumulated high levels of both erythroblast and c-Kit^+^Mac-1^lo^ myeloid blasts in BM and spleen (Figures 2C, 2D and S4E–S4H), replicating the original disease phenotypes. Therefore, biallelic *Cebpa* and *Gata2* ZnF1 mutations in combination, but not separately, are able to induce highly aggressive, transplantable bilineage AEL.

### Identification of the AEL-Sustaining Leukemia-Initiating Cell

**Figure 3:**
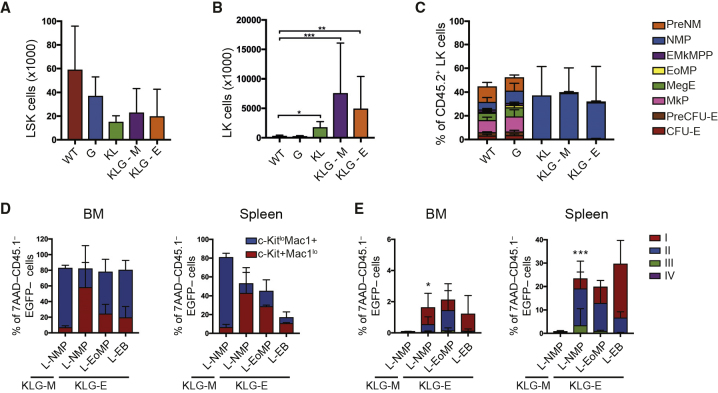
*Cebpa* and *Gata2* Mutant AEL Is Sustained by LICs with an NMP Immuno-Phenotype (A) Absolute number of LSK in the BM of terminal analyzed primary transplanted mice of the indicated genotypes. The results are presented as the mean ± SD. Statistical significance was determined using the Mann-Whitney U test. ^∗^p < 0.05, ^∗∗^p < 0.01, ^∗∗∗^p < 0.001. WT, n = 7; G, n = 9; KL, n = 4; KLG-M, n = 4; KLG-E, n = 3 from a total of five independent experiments. (B) Absolute number of LK cells in the BM analyzed as in (A). The results are presented as the mean ± SD. WT, n = 7; G, n = 9; KL, n = 5; KLG-M, n = 8; KLG-E, n = 5 in five independent experiments. (C) Myelo-erythroid progenitors as a percentage of donor LK cells in the BM in mice from (B). The results are presented as the mean ± SD. (D) Terminal analysis of secondary recipients transplanted with purified L-NMPs, L-EoMPs, and L-EB cells. Histogram showing c-Kit^lo^Mac1^+^ and c-Kit^+^Mac1^lo^ cells as a percentage of 7AAD^–^CD45.1^–^EGFP^–^ cells in the BM (left panel) and spleen (right panel). The results are presented as the mean ± SD. KLG-M L-NMP, n = 2; KLG-E L-NMP, n = 5; KLG-E L-EoMP, n = 2; KLG-E L-EB, n = 2 in three independent experiments. (E) Histogram showing stage I–IV erythroblast cells as a percentage of 7AAD^–^CD45.1^–^EGFP^–^ cells in the BM (left panel) and spleen (right panel) in mice from (D). The results are presented as the mean ± SD. ^∗^p < 0.05, ^∗∗∗^p < 0.005 (combined stage I and II EB; Student's t test, compared with KLG-M L-NMP). PreNM, pre-neutrophil-monocyte progenitor; EMkMPP, erythroid-megakaryocyte primed multi-potent progenitor; MegE, megakaryocyte erythroid progenitor; PreCFU-E, pre-colony forming unit erythroid progenitor; CFU-E, colony forming unit erythroid progenitor See also Figures S5–S7 and Tables S2 and S3.

To determine if erythroid and myeloid AEL blasts arose from the same leukemia-initiating cell (LIC) we examined the CD45.2 stem and progenitor cell compartment in leukemic mice to identify a putative LIC population(s). We did not observe any expansion of the BM CD45.2 Lin^–^Sca-1^+^c-Kit^+^ (LSK) stem- and multi-potent progenitor compartment in leukemic mice (Figure 3A). In contrast, the BM CD45.2 Lin^–^c-Kit^+^ (LK) population was significantly expanded in leukemic compared with non-leukemic mice (Figure 3B). Using our recently described progenitor phenotyping scheme (Drissen et al., 2016) (Figures S5A–S5C) we found that CD45.2^+^ LK cells from non-leukemic WT and G mice displayed a normal distribution of myelo-erythroid progenitors (Figure 3C). In contrast, in leukemic mice the LK compartment consisted principally of LKCD41^–^CD150^–^FcγRII/III^+^CD55^–^ cells (Figure 3C), the immuno-phenotype of NMPs (Figure S5B). We also observed a significant amount of LKCD41^+^CD150^–^ cells in leukemic mice. Normally, these cells are rare and phenotypically heterogeneous (Figure S6A). However, in leukemic mice they were abundant and predominantly FcγRII/III^+^CD55^–^, similar to NMPs, with a small FcγRII/III^+^CD55^+^ population observed selectively in KLG-E leukemias (Figure S6A). We therefore defined leukemic NMPs (L-NMPs) as LKFcγRII/III^+^CD55^–^ (Figure S6B), thereby including both the CD41^+^ and CD41^–^ cell populations. From KLG-E mice we also purified LKFcγRII/III^+^CD55^+^ cells (designated L-EoMPs, based on their phenotypic similarity to the previously defined eosinophil-mast cell progenitor) (Drissen et al., 2016) (Figures S5A–S5C) and CD45^–^Lin^–^c-Kit^+^ cells (designated L-EB, as they have the surface phenotype of the c-Kit^+^ stage I erythroblast identified above) (Figure S6B). Transplantation of purified L-NMPs from either KLG-M or KLG-E mice, or KLG-E L-EoMPs or L-EBs, in all cases re-capitulated the phenotype of the original disease (Figures 3D, 3E, and S7A–S7H; Table S2). LIC titration experiments showed comparable engraftment of KLG L-NMP and L-EoMP, with L-EBs significantly lower (Table S3). Given the far greater abundance of L-NMPs compared with L-EoMPs (Figures 3C and S6B), the main LIC population in both KLG-M and KLG-E mice was the L-NMP. Furthermore, KLG-E LICs could re-establish both transformed erythroid and myeloid cells in secondary recipients.

### Erythroleukemic L-EBs Show Ectopic Myeloid Transcriptional Programming

**Figure 4:**
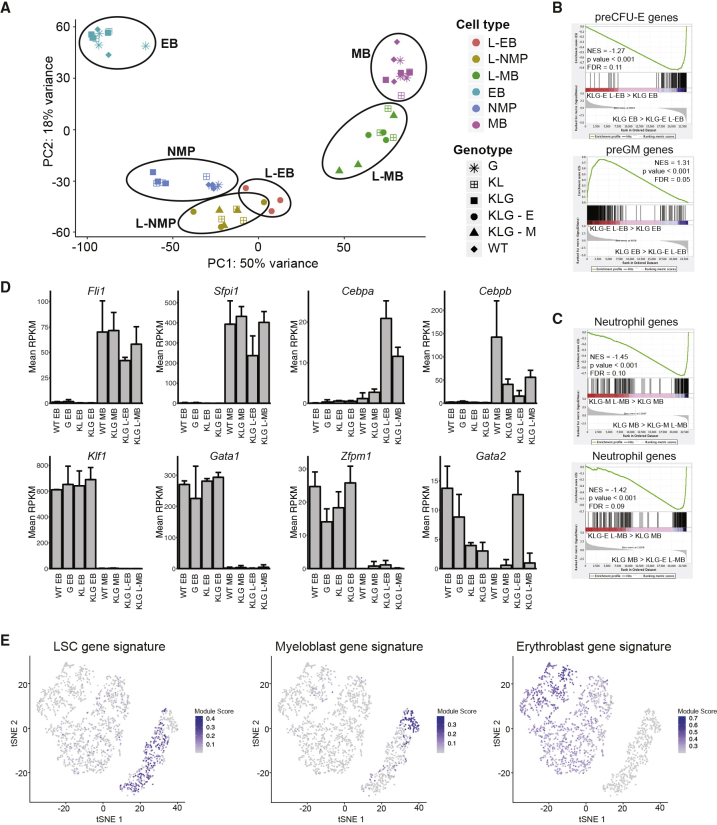
Erythroid Leukemia LICs Show Ectopic Myeloid Transcriptional Programming (A) Principal-component analysis of RNA sequencing data using the top 500 most variable genes across the entire dataset. The ovals have been drawn to encompass the populations indicated next to them, n = 3 per population. (B) GSEA of KLG-E L-EB versus KLG EB using preCFU-E (top panel) and preGM gene sets (bottom panel). Normalized enrichment score (NES), p value and false discovery rate (FDR) are indicated. (C) GSEA of KLG-M L-MB versus KLG MB (top panel) and KLG-E L-MB versus KLG MB (bottom panel) using a neutrophil differentiation-specific gene set as in (B). (D) Histograms showing expression levels of selected TF-encoding genes measured by RNA sequencing in the indicated cell populations. Values are mean reads per kilobase million (RPKM) ± SD, n = 3 per population. (E) tSNE plots of human AEL single cell showing expression of indicated signatures. See also Figure S8 and Table S4.

Both normal/pre-leukemic and leukemic progenitors were RNA sequenced. Clustering using principal components showed that non-leukemic MBs, EBs, and NMPs clustered according to cell identity (Figure 4A). The leukemic MB (L-MB) and L-EB populations clustered closer to the NMP, consistent with a more immature, progenitor-like state. Using gene set enrichment analysis (GSEA) (Subramanian et al., 2005) we observed that erythroid differentiation-specific genes were downregulated in KLG L-EBs compared with pre-leukemic KLG EBs, whereas myeloid gene expression was upregulated (Figure 4B). In addition, expression of neutrophil differentiation-specific genes was lower in KLG-M and KLG-E L-MBs compared with pre-leukemic KLG MBs (Figure 4C). Therefore, the block in morphological differentiation along the erythroid and neutrophil lineages was accompanied by, and likely due to, suppression of the respective differentiation programs at the molecular level. Examination of the genes differentially expressed between KLG L-EB and pre-leukemic EBs (Table S4) identified *Cebpa*, *Cebpb*, *Fli1*, and *Sfpi1* encoding, in addition to C/EBPα, the C/EBPβ, FLI-1, and PU.1 TFs, respectively, as highly upregulated to the levels observed in normal (WT MB) and transformed myeloid blasts (KLG-E L-MB, KLG-M L-MB) (Figures 4D and S8A), whereas *Gata1*, *Klf1*, and *Zfpm1* (encoding FOG-1), all genes encoding TFs critical to erythroid development, were strongly downregulated in L-EBs (Figures 4D and S8A). In contrast, *Gata2* expression was sustained in L-EBs at the same level as in WT EBs (Figures 4D and S8A). The differentiation block of L-EBs is therefore accompanied by the expression of several TFs normally absent in erythroid lineage cells.

To assess if the *Cebpa* and *Gata2* mutant mouse model was comparable with human AEL we performed flow cytometry of human AEL patient samples, observing the presence of both myeloid (CD33^+^) and erythroid (CD71^+^CD235a^+^) blasts, as well as an expanded CD71^–^CD235a^–^CD33^+^KIT^+^CD34^+^ myeloid progenitor population (Figure S8B). Single-cell RNA sequencing and tSNE-based clustering identified AEL cell populations expressing human MB, erythroblast, and AML leukemic stem cell (LSC) gene signatures (Figure 4E), and showed that the LSC-like population was identified by the same markers as the expanded CD71^–^CD235a^–^CD33^+^KIT^+^CD34^+^ progenitor subset, whereas cells expressing the MB and erythroblast signatures expressed *CD33*, and *TFRC* and *GYPA* (which encode CD71 and CD235a), respectively, consistent with the flow cytometry data (Figures S8C and S8D; *KIT* expression not detected in 10× data). Finally, using GSEA a human AEL-specific gene signature was upregulated in KLG-E compared with KLG-M L-NMPs (Figure S8E). By both cellular and molecular criteria the murine AEL model is therefore analogous to human AEL, and in particular an expanded myeloid progenitor population with LSC characteristics, analogous to the L-NMP, could be identified in human AEL samples.

### Biallelic *Cebpa* Mutant NMPs Display Ectopic Erythroid Lineage Programming

**Figure 5:**
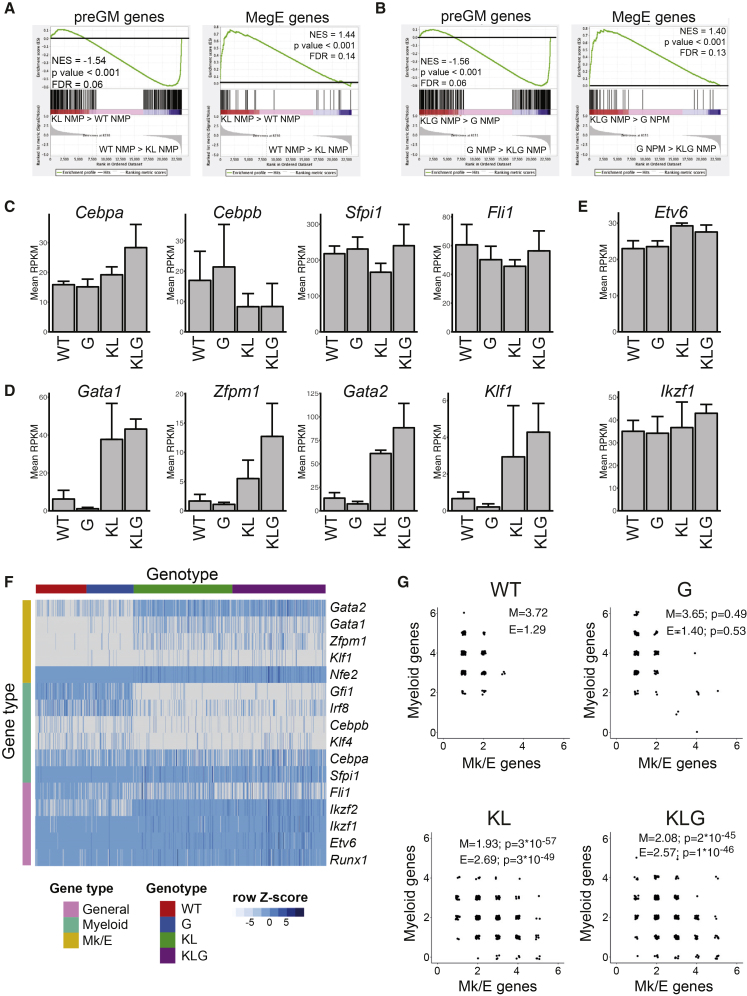
Biallelic *Cebpa* Mutations Install Ectopic Erythroid Lineage Programming in NMPs (A) GSEA of KL NMPs versus WT NMPs using pre-granulocyte-macrophage progenitor (preGM) (left panel) and MegE gene sets (right panel). NES, p value and FDR are indicated. n = 3 per genotype. (B) GSEA of KLG NMPs versus G NMPs using preGM (left panel) and MegE gene sets (right panel). n = 3 per genotype. (C) Histogram showing expression levels of selected myeloid TF-encoding genes measured by RNA sequencing in NMPs of the indicated genotypes. Values are mean RPKM ± SD, n = 3 per genotype. (D) Histogram showing expression levels of selected erythroid TF-encoding genes, as in (C). (E) Histogram showing expression levels of selected general hematopoietic TF-encoding genes, as in (C). (F) Multiplex qRT-PCR of myeloid and megakaryocytic/erythroid (Mk/E) TF genes on single NMPs. WT, n = 192; G, n = 192; KL, n = 384; KLG, n = 384. The heatmap shows 2^–ΔCt^ values normalized to *Hprt* and centered on the mean value for each gene. (G) Scatterplot depicting the number of myeloid and Mk/E TF genes from (F) co-expressed in single WT, G, KL, and KLG NMPs. Each dot represents a single cell. The average number of myeloid (M) and Mk/E genes expressed is shown, as are the p values (Wilcox test) against the WT distribution for each gene set. See also Table S4.

NMPs normally do not have detectable erythroid lineage potential (Drissen et al., 2016). However, we previously observed that pre-leukemic HSCs from KL mice were enriched for erythroid gene expression compared with their WT counterparts (Bereshchenko et al., 2009). To determine if a similar effect was present in *Cebpa* mutant progenitors we compared the gene expression profiles of pre-leukemic NMPs from the four genotypes (Table S4). Comparing WT and KL NMPs we observed depletion of myeloid and enrichment of megakaryocyte-erythroid gene expression (Figure 5A) in the KL mutant NMPs. The same pattern was observed comparing G with KLG NMPs (Figure 5B). To assess the underlying transcriptional reprogramming we analyzed the expression of key myeloid (*Cebpa*, *Cebpb*, *Fli1*, and *Sfpi1*) and erythroid (*Gata1*, *Gata2*, *Klf1*, and *Zfpm1*) TF-encoding genes, along with those encoding more generally expressed hematopoietic TFs (*Ikzf1*, *Etv6*, and *Runx1*) in the RNA sequencing dataset. Although the myeloid TFs showed moderate or no regulation (Figure 5C), erythroid TFs were upregulated in NMPs in the presence of biallelic *Cebpa* mutation (Figure 5D), with little change seen for *Etv6* or *Ikzf1* (Figure 5E). To determine if the upregulated erythroid TFs were co-expressed with myeloid TFs at the single-cell level we performed microfluidics-based qRT-PCR (Figure 5F). This confirmed the observations from bulk RNA sequencing, and showed that, while WT and G NMPs expressed multiple myeloid TFs, the expression of multiple erythroid TFs was rare (Figure 5G using genes from Figure 5F). In contrast, in the presence of biallelic *Cebpa* mutation NMPs consistently co-expressed myeloid and erythroid TFs (Figure 5G). This analysis showed that, in the presence of the KL genotype the frequency of erythroid TF expression was increased, whereas myeloid TFs, while still expressed, were present at lower frequencies. The expression of *Ikzf1* and *Etv6* was not affected by *Cebpa* mutation (Figure 5F), consistent with the RNA sequencing data.

### *Gata2* ZnF1 Mutation Promotes Erythroid and Restricts Myeloid TF Chromatin Access

**Figure 6:**
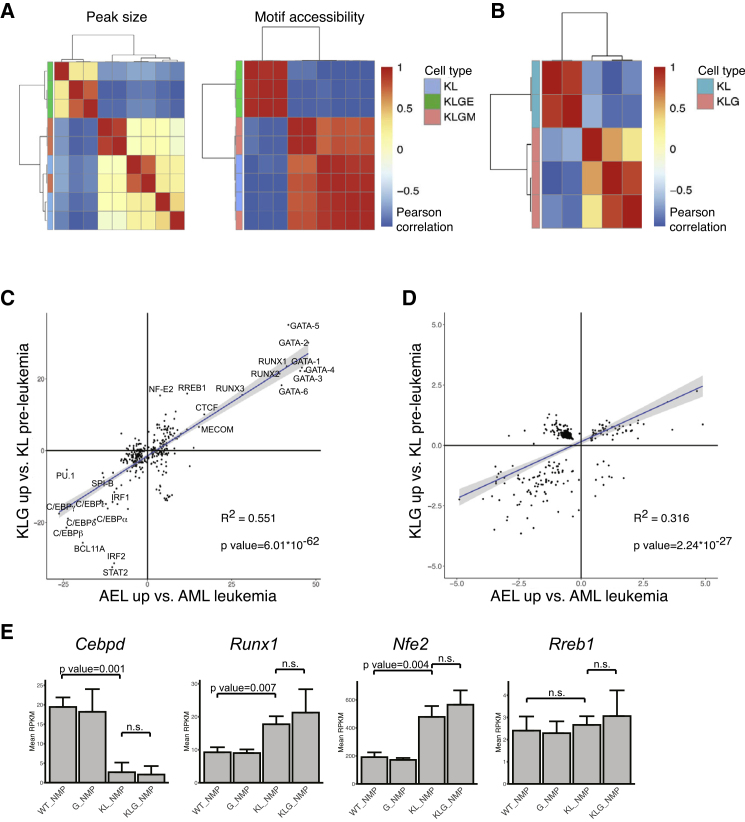
Mutation of GATA-2 ZnF1 Induces an Erythroid-Permissive Chromatin State (A) Leukemic KL, KLG-M, and KLG-E L-NMP were hierarchically clustered using Pearson correlation of ATAC sequencing peak intensities (left panel) and motif accessibility (right panel). n = 3 per genotype. (B) Pre-leukemic KL (n =3) and KLG NMP (n = 2) samples were hierarchically clustered using Pearson correlation of motif accessibility. (C) Plot showing linear modeling of the correlation between TF motifs with significantly different accessibility in AEL versus AML L-NMPs, samples from (A, x axis) and KL versus KLG NMPs, samples from (B, y axis). The linear model and associated R^2^ and p values are shown. (D) Plot showing linear modeling of the correlation between promoters with significantly different accessibility as in (C). (E) Expression of genes encoding cognate TFs for correlated motifs from (C) measured as in Figures 5C–5E. TFs already included in Figures 5C–5E are not shown. Values are mean RPKM ± SD, n = 3 per genotype. See also Tables S5 and S6.

Although biallelic *Cebpa* mutation upregulated erythroid TFs, we only observed AEL in KLG mice, indicating an additional layer of regulation imposed by *Gata2* ZnF1 mutation. Exome sequencing of KLG-E and KLG-M tumors did not identify any distinct, recurring coding sequence mutations (Table S5), arguing against additional genetic drivers being involved. We therefore performed ATAC sequencing of purified KL, KLG-M, and KLG-E L-NMPs to assess whether these were epigenetically distinct. Clustering based on peak intensity or TF motif chromatin accessibility (Figure 6A; Table S6) clearly separated KL and KLG-M from KLG-E L-NMPs. Motif-based clustering also separated pre-leukemic KL and KLG NMPs (Figure 6B; Table S6), and we observed a clear correlation of motif-enrichment in leukemic and pre-leukemic samples: in both KLG-E L-NMPs and KLG NMPs chromatin access to erythroid TF motifs (GATA, NF-E2, and RREB) was increased, whereas access to myeloid TF motifs (C/EBP, PU.1, and SPI-B) was decreased (Figure 6C). Access to individual promoters was similarly correlated (Figure 6D). However, the expression level of the cognate TF-encoding genes was not different between KL and KLG NMPs (Figures 5C, 5D, and 6E). The *Gata2* G320D mutation therefore generates an erythroid-permissive chromatin state in pre-leukemic NMPs, without altering the expression of erythroid or myeloid TFs, a chromatin state that is preserved upon their transformation to KLG-E L-NMPs.

**Figure 7:**
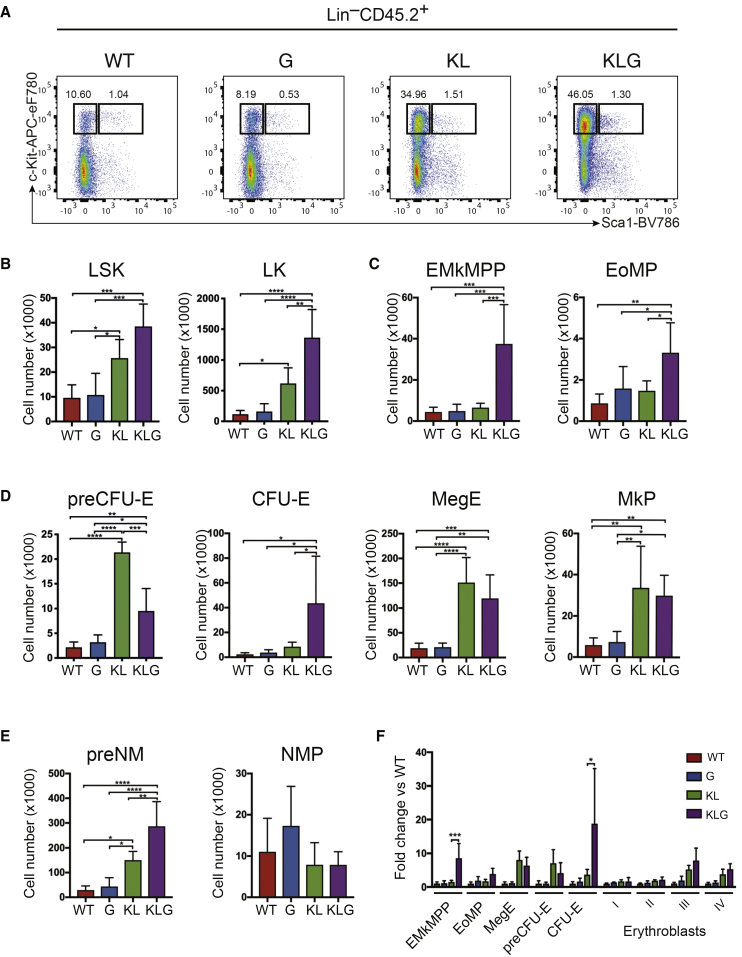
Mutation of GATA-2 ZnF1 Impairs Differentiation at Distinct Stages on Myelo-Erythroid Differentiation (A) Representative FACS plots LSK and LK cells in the BM in pre-leukemic mice 6 weeks post-transplantation. (B) Absolute number of LSK (left panel) and LK cells (right panel) in the BM in mice from (A). (C) Absolute number of phenotypic EMkMPP (left panel) and EoMP progenitors (right panel) in the BM in mice from (A). (D) Absolute number of phenotypic committed erythroid/megakaryocytic progenitors in the BM in mice from (A). (E) Absolute number of phenotypic committed neutrophil-monocyte progenitors in the BM in mice from (A). (F) Number of myelo-erythroid progenitors from (C and D) and stage I–IV erythroblasts normalized to WT values in mice from (A). Myelo-erythroid progenitor analysis was performed on five to six replicates from two independent experiments. Stage I–IV erythroblast analysis was performed on three to four replicates from one experiment. The results were analyzed using a multiple comparison ANOVA. The results are presented as the mean ± SD. ^∗^p < 0.05, ^∗∗^p < 0.01, ^∗∗∗^p < 0.001, ^∗∗∗∗^p < 0.0001.

To assess the effect of the transcriptional and epigenetic changes induced by *Cebpa* and *Gata2* mutation on lineage commitment we analyzed pre-leukemic BM progenitors 6 weeks post-transplantation (Figure S5C), before any increase in myeloid cell output in KLG mice. Both the LSK and LK populations were increased by *Cebpa* mutation (Figures 7A and 7B), and the most significant expansion was of *Gata1*-expressing myelo-erythroid progenitors, and in particular those with erythroid and megakaryocytic lineage potential; EMkMPPs, MegEs, MkPs, PreCFU-Es and CFU-Es (Figures 7C–7E), providing a cellular mechanism for the more rapid reconstitution of erythrocytes by KL and KLG FL cells after transplantation (Figure S2G). By normalizing the size of the progenitor populations to that of WT mice we observed that EMkMPPs and CFU-Es were selectively expanded in KLG compared with KL mice (Figure 7F), demonstrating a co-operative effect of the two mutations on the progenitor hierarchy, and in particular in the generation of committed erythroid CFU-E progenitors.

### Pre-leukemic NMPs and Erythroleukemic KLG L-NMPs Are Bipotent at the Single-Cell Level

**Figure 8:**
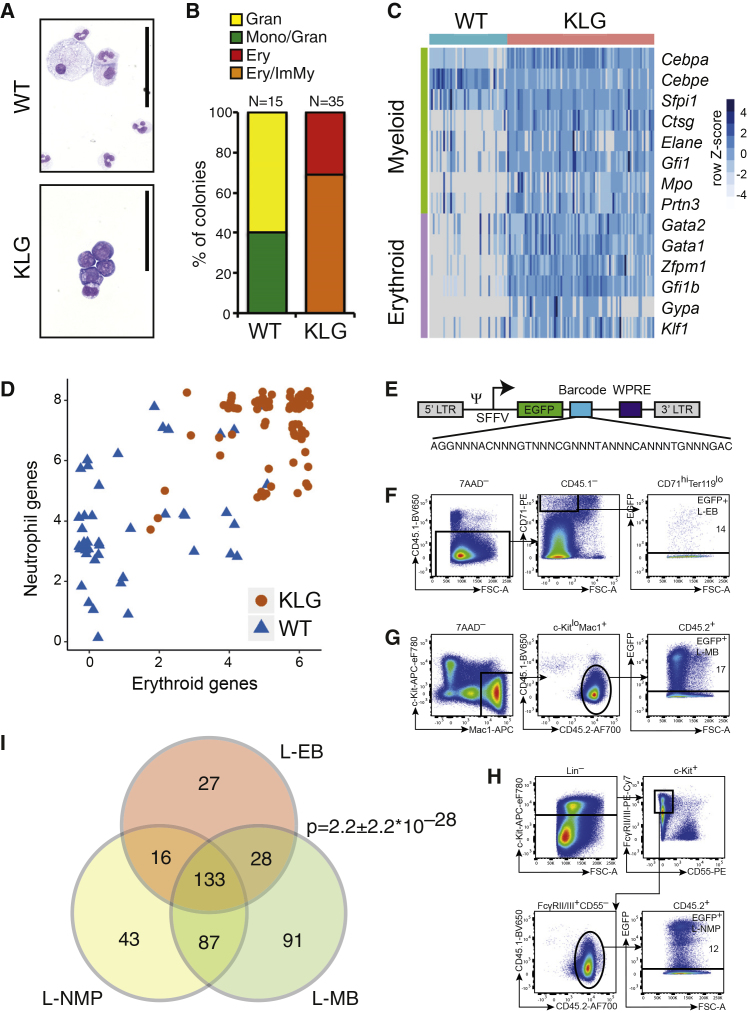
AEL LICs Are Bipotent at the Single-Cell Level (A) Cytospins of single NMP colonies stained with May-Grünwald and Giemsa. Scale bars, 50 μm. (B) The morphology of colonies generated from single WT and KLG NMPs is shown. Gran, granulocytic; Mono, monocytic; Ery, erythroid; ImMy, immature myeloid. The total number of colonies scored for each genotype is indicated. (C) Multiplex qRT-PCR of myeloid and erythroid genes on colonies derived from single NMPs. WT, n = 45; KLG, n = 85. The heatmap shows 2^–ΔCt^ values normalized to the average of *Gapdh* and *Hprt* and centered on the mean value for each gene. (D) Scatterplot depicting the number of myeloid and erythroid genes co-expressed in individual WT and KLG colonies from (C). (E) Schematic of the lentiviral barcoded library vector. (F) Sorting strategy for re-isolation EGFP^+^ L-EBs from mice transplanted with barcoded KLG-E L-NMPs. Data representative of three independent transplantation experiments are shown. Percentages of re-isolated transduced cells are indicated. (G) Sorting strategy for re-isolation of EGFP^+^ L-MBs as in (F). (H) Sorting strategy for re-isolation of EGFP^+^ L-NMPs as in (F). (I) Venn diagram depicting the overlap of barcodes retrieved from the populations isolated above (F–H). Data are representative of three independent transplantation experiments. Mean p value ± SD of three independent transplantations is shown (hypergeometric test).

These data were compatible with *Cebpa* and *Gata2* mutation co-operating to install erythroid lineage potential in NMPs. We therefore cultured single WT and KLG NMPs under conditions compatible with both myeloid and erythroid lineage development, and assessed their differentiation by both morphology and gene expression. As expected, WT NMPs generated cells with neutrophil and monocyte morphology (Figures 8A and 8B) and predominantly myeloid gene expression (ratio of erythroid [*Gata1*, *Gata2*, *Zfpm1*, *Gfi1b*, *Gypa*, and *Klf1*] to neutrophil [*Cebpa*, *Cebpe*, *Ctsg*, *Elane*, *Mpo*, *Prtn3*, *Sfpi1*, and *Gfi1*] gene expression frequency: 0.41) (Figures 8C and 8D). In contrast, KLG NMPs generated colonies containing immature myeloid and erythroid cells (Figures 8A and 8B), with the immature myeloid morphology in KLG colonies likely due to the increased proliferative capacity of myeloid progenitors after loss of C/EBPα-mediated E2F repression (Porse et al., 2005). KLG colonies expressed erythroid genes at significantly higher frequency compared with WT NMP colonies (ratio of erythroid to myeloid gene expression frequency: 0.97; p value versus WT = 8.6 × 10^−9^), and consistently co-expressed erythroid and neutrophil lineage-specific genes demonstrating the generation of both neutrophil and erythroid lineage cells from a single KLG NMP (Figures 8C and 8D). KLG NMPs therefore represent a neomorphic progenitor population capable of efficiently generating both neutrophil and erythroid lineage cells, replicating the lineage pattern observed in KLG erythroleukemic mice.

The observation that pre-leukemic KLG NMPs were bipotent neutrophil-erythroid progenitors, raised the possibility that L-NMPs were also bipotent, and generated both myeloid and erythroid blasts at the single-cell level. To test this hypothesis we isolated KLG L-NMPs from KLG-E mice, transduced them with a lentiviral barcode library containing 725 barcodes, contained in an EGFP-expressing viral backbone (Figure 8E) (Belderbos et al., 2017), and transplanted the transduced cell population into irradiated recipients. After 4 weeks we re-isolated EGFP-expressing L-NMPs, L-EBs, and L-MBs (Figures 8F–8H), retrieved the barcodes from their genomic DNA, and identified them by next-generation sequencing. By comparing the barcodes retrieved from L-EBs and L-MBs we found that there was a highly significant overlap in three independent transplantations (Figure 8I), demonstrating that the transplanted L-NMPs remain bipotent after transformation. Importantly, the number of barcodes retrieved from all three populations was significantly higher than randomly expected (p < 0.00007 in all three experiments), consistent with the barcoded L-NMPs self-renewing and at the same time generating both L-MB and L-EB blasts. Together, these results therefore show that KLG NMPs retain their neomorphic neutrophil-erythroid lineage potential after leukemic transformation, allowing individual L-NMPs to propagate the disease and to generate both transformed myeloid and transformed erythroid blasts.

## Discussion

We here show that biallelic *Cebpa* and *Gata2* ZnF1 mutations cooperate during myeloid leukemogenesis, and in particular that these mutations are sufficient to induce bilineage AEL. Our murine AEL model resembles human AEL, containing both myeloid and erythroid blasts, the cardinal feature of bilineage AEL. In addition, the major LIC population in the murine AEL model has an NMP immune-phenotype, and we identify a corresponding expanded CD33^+^CD34^+^KIT^+^ myeloid progenitor in human AEL, which expressed a human AML LSC signature.

The L-NMPs capable of initiating bilineage AEL are bipotent at the single-cell level. This L-NMP is similar to that sustaining *Cebpa* mutant neutrophil lineage leukemia (Bereshchenko et al., 2009, Kirstetter et al., 2008); however, while, NMPs normally generate only neutrophils and monocytes (Drissen et al., 2016), in the presence of both biallelic *Cebpa* and *Gata2* ZnF1 mutations they display ectopic erythroid differentiation potential, as well as the capacity to generate bilineage L-NMPs. Here, we find that *Cebpa* and *Gata2* mutations make distinct contributions to erythroid lineage programming of NMPs: biallelic *Cebpa* mutation increases the expression of erythroid lineage TFs, while *Gata2* ZnF1 mutation increases erythroid TF and decreases myeloid TF chromatin access. This erythroid-permissive chromatin state is sustained in bilineage KLG-E L-NMPs, but not myeloid-only KLG-M L-NMPs, further supporting its role in maintaining the bilineage AEL phenotype.

Genetic alterations affecting chromatin regulators are present in the majority of AML tumors, with *DNMT3A* and *TET2* mutations the most common (Metzeler et al., 2016). In genetic modeling such mutations have been shown to de-regulate methylation of both tumor suppressor (Rasmussen et al., 2015) and differentiation-specific enhancers (Yang et al., 2016), and in the case of *DNMT3A* to control the lineage identity of the resulting leukemia (Yang et al., 2016). We here identify GATA-2 as a “non-canonical” chromatin regulator that is able to selectively control access to lineage-specific TFs motifs, thereby controlling the phenotype of the resulting leukemia. This is consistent with GATA-2 physically and functionally interacting with both myeloid (PU.1 and C/EBP) and erythroid TFs (KLF1, FOG-1, and SCL/LMO2/LDB1) (Collin et al., 2015), and altered crosstalk within this TF network upon *Gata2* ZnF1 mutation contributing to chromatin reorganization.

The mechanisms underlying erythroid lineage transformation in AEL remain unknown. We here find that transformed L-EBs upregulate a number of genes encoding myeloid lineage TFs, including *Fli1* and *Sfpi1*. Overexpression of both these genes through retroviral insertion induces pure erythroid leukemia (Ben-David et al., 1990, Moreau-Gachelin et al., 1988), and their continued expression is necessary and sufficient to block erythroid differentiation of transformed erythroblasts (Rao et al., 1997, Starck et al., 1999). Importantly, FLI-1 and PU.1 cross-antagonize the key erythroid TFs GATA-1, GATA-2, and KLF-1: PU.1 is able to suppress GATA-1 both transcriptionally (Nerlov and Graf, 1998) and through protein-protein interaction (Rekhtman et al., 1999), and FLI-1 inhibits KLF1-mediated transcription (Starck et al., 2003). Therefore, the sustained expression of FLI-1 and PU.1 in L-EBs can explain the absence of both KLF-1 and GATA-1 expression, and the observed differentiation block. Importantly, PU.1–GATA inhibition is reciprocal, as GATA-1 and GATA-2 also block PU.1 function (Nerlov et al., 2000, Zhang et al., 2000). Therefore, sustained expression of GATA-2 in L-EBs, in conjunction with decreased chromatin access of myeloid TFs, may prevent their conversion to myeloid lineage cells, despite the extensive myeloid transcriptional reprogramming of L-EBs.

In summary, we here identify combined *Cebpa* and *Gata2* mutations as causative of bilineage AEL, providing a validated pre-clinical model for this leukemia subtype. In addition, we identify a previously uncharacterized role of *Gata2* ZnF1 in controlling lineage fate through modification of TF chromatin access. The loss of myeloid and gain of erythroid TF chromatin access in the presence of *Gata2* ZnF1 mutation may be relevant to the myeloid differentiation block characteristic of AML, and in particular act cooperatively with altered TF gene expression induced by biallelic *Cebpa* mutation, providing a molecular basis for the correlation of *CEBPA* and *GATA2* mutation in AML. These studies underscore the usefulness of accurate genetic modeling and the study of the pre-leukemic state in understanding the etiology of AML.

## STAR★Methods

### Key Resources Table

**Table:** 

REAGENT or RESOURCE	SOURCE	IDENTIFIER
**Antibodies**		

7-Aminoactinomycin D (7AAD)	Cayman Chemical	Cat#11397
Anti-mouse CD4 APC-eF780	eBioscience	Clone RM4-5, Cat#47-0042-82; RRID: AB_1272183
Anti-mouse CD8a APC-eF780	eBioscience	Clone 53-6.7, Cat#47-0081-82; RRID: AB_1272185
Anti-mouse NK1.1 PB	BioLegend	Clone PK136, Cat#108722; RRID: AB_2132712
Anti-mouse Gr1 PO	ThermoFisher	Clone RB6-8C5, Cat#RM3030; RRID: AB_2556571
Anti-mouse CD19 PE-Cy7	ThermoFisher	Clone 1D3, Cat#25-0193-82; RRID: AB_657663
Anti-mouse Mac1 APC	BioLegend	Clone M1/70, Cat#101212; RRID: AB_312795
Anti-mouse CD45.1 PE	eBioscience	Clone A20, Cat#12-0453-83; RRID: AB_465676
Anti-mouse CD45.2 AF700	BioLegend	Clone 104, Cat#109822; RRID: AB_493731
Anti-mouse CD4 PE-Cy5	BioLegend	Clone RM4-5, Cat#100514; RRID: AB_312717
Anti-mouse CD8a PE-Cy5	BioLegend	Clone 53-6.7, Cat#100710; RRID: AB_312749
Anti-mouse Ter119 PE-Cy5	BioLegend	Clone TER-119, Cat#116210; RRID: AB_313711
Anti-mouse Mac1 PE-Cy5	BioLegend	Clone M1/70, Cat#101210; RRID AB_312793
Anti-mouse Gr1 PE-Cy5	BioLegend	Clone RB6-8C5 Cat#108410; RRID: AB_313375
Anti-mouse CD150 APC	BioLegend	Clone TC15-12F12.2, Cat#115910; RRID: AB_493460
Anti-mouse c-Kit APC-eF780	eBiosciences	Clone 2B8, Cat#47-1171-82; RRID: AB_1272177
Anti-mouse CD45.1 BV650	BioLegend	Clone A20, Cat#110736; RRID: AB_2562564
Anti-mouse CD48 APC	BioLegend	Clone HM48-1, Cat#103412; RRID: AB_571997
Anti-mouse CD150 PE-Cy7	BioLegend	Clone TC15-12F12.2, Cat#115914; RRID: AB_439797
Anti-mouse Sca1 PB	BioLegend	Clone D7, Cat#108120; RRID: AB_493273
Streptavidin PE-Texas Red	BD	Cat#551487; RRID: AB_10054235
Anti-mouse Flt3 PE	BioLegend	Clone A2F10, Cat#135306; RRID: AB_1877217
Anti-mouse CD5 PE-Cy5	BioLegend	Clone 53-7.3, Cat#100610; RRID: AB_312739
Anti-mouse B220 PE-Cy5	BioLegend	Clone RA3-6B2, Cat#103210; RRID: AB_312995
Anti-mouse FcγRII/III PE-Cy7	eBioscience	Clone 93, Cat#25-0161-82; RRID: AB_469598
Anti-mouse Sca1 BV605	BioLegend	Clone D7, Cat#108133; RRID: AB_2562275
Anti-mouse CD105 Biotin	eBioscience	Clone MJ7/18, Cat#13-1051-85; RRID: AB_466557
Anti-mouse CD41 BV421	BioLegend	Clone MWReg30, Cat#133911; RRID: AB_10960744
Anti-mouse CD55 PE	BioLegend	Clone RIKO-3, Cat#131804; RRID: AB_1279265
Anti-mouse CD71 PE	BioLegend	Clone RI7217, Cat#113808; RRID: AB_313569
Anti-mouse PerCP-Cy5.5	eBioscience	Clone TER-119, Cat#45-5921-82; RRID: AB_925765
Anti-mouse CD41 PE	eBioscience	Clone MWReg30, Cat#12-0411-83; RRID: AB_763486
Anti-human CD3 BV421	BioLegend	Clone OKT3, Cat#317343; RRID: AB_2565848
Anti-human CD4 BV421	BioLegend	Clone OKT4, Cat#317433; RRID: AB_11150413
Anti-human CD8a BV421	BioLegend	Clone RPA-T8, Cat#301035; RRID: AB_10898322
Anti-human CD10 PE-Cy5	BioLegend	Clone HI10a, Cat#312206; RRID: AB_314917
Anti-human CD19 PE-Cy5	BioLegend	Clone HIB19, Cat#302210; RRID: AB_314240
Anti-human CD20 PE-Cy5	BioLegend	Clone 2H7, Cat#302308; RRID: AB_314256
Anti-human CD56 PE-Cy5	BioLegend	Clone MEM-188, Cat#304608; RRID: AB_314450
Anti-human CD71 FITC	BioLegend	Clone CY1G4, Cat#334104; RRID: AB_2201482
Anti-human CD235ab APC	BioLegend	Clone HIR2, Cat#306608; RRID: AB_314626
Anti-human CD117 APC-Fire750	BioLegend	Clone 104D2, Cat#313240; RRID: AB_2632949
Anti-human CD33 PE	BioLegend	Clone P67.6, Cat#366608; RRID: AB_2566107
Anti-human CD34 AF700	BioLegend	Clone 581, Cat#34352; RRID: AB_2561495
Anti-human CD38 PE-TexasRed	ThermoFisher	Clone HIT2, Cat#MHCD3817; RRID: AB_10392545
Anti-human CD3 BV421	BioLegend	Clone OKT3, Cat#317343; RRID: AB_2565848
Anti-human CD4 BV421	BioLegend	Clone OKT4, Cat#317433; RRID: AB_11150413
See Table S8		

**Bacterial and Virus Strains**		

pEGZ2-linkerBC322 barcoding library	(Belderbos et al., 2017)	

**Biological Samples**		

AEL patient samples (OX1164; AYL050; MKH048; STB115)	MDSBio	NA
Normal adult human bone marrow	AllCells	NA

**Critical Commercial Assays**		

CellDirect One-Step qPT-PCR kit	ThermoFisher	Cat#11753100
Biomark 192.24 Gene Expression IFCs	Fluidigm	Cat#101-0351
Nextera XT Index Kit	Illumina	Cat#FC-131-1001
PEIpro	Polyplus transfection	Cat#115-100

**Deposited Data**		

Raw and analyzed data	This paper	GEO: GSE141813
Human M6 AEL gene expression data	(Taskesen et al., 2015) (Taskesen et al., 2011) (Wouters et al., 2009)	GEO: GSE14468

**Experimental Models: Cell Lines**		

HEK293T/17 cells	ATCC	Cat#ATCC CRL-11268

**Experimental Models: Organisms/Strains**		

Mouse: CD45.1/CD45.1-*Gata1*-EGFP	(Drissen et al., 2016)	NA
Mouse: *Cebpa*^*K/L*^*;Gata2*^*D/+*^	This paper, (Bereshchenko et al., 2009) (Kirstetter et al., 2008)	NA

**Oligonucleotides**		

See Table S7		

**Software and Algorithms**		

Flowjo	FlowJo LLC	RRID:SCR_008520
FastQC	Babraham Bioinformatics	http://www.bioinformatics.babraham.ac.uk/projects/fastqc; RRID:SCR_014583
STAR	(Dobin et al., 2013)	https://github.com/alexdobin/STAR/releases; RRID:SCR_015899
featureCounts	(Liao et al., 2013)	http://subread.sourceforge.net/; RRID:SCR_012919
DESeq2	(Love et al., 2014)	https://bioconductor.org/packages/release/bioc/html/DESeq2.html; RRID:SCR_015687
GSEA	(Subramanian et al., 2005)	http://software.broadinstitute.org/gsea/index.jsp; RRID:SCR_003199
Cell Ranger	10x Genomics	https://support.10xgenomics.com/single-cell-gene-expression/software/overview/welcome; RRID:SCR_017344
Seurat	(Butler et al., 2018)	https://satijalab.org/seurat/; RRID:SCR_016341
Trim Galore	Babraham Bioinformatics	https://github.com/FelixKrueger/TrimGalore; RRID:SCR_016946
Bowtie2	(Langmead and Salzberg, 2012)	http://bowtie-bio.sourceforge.net/bowtie2/index.shtml; RRID_005476
Samtools	(Li et al., 2009)	http://www.htslib.org/; RRID:SCR_002105
Picard	Broad Institute	https://broadinstitute.github.io/picard/; RRID:SCR_006525
MACS2	(Zhang et al., 2008)	https://github.com/taoliu/MACS; RRID:SCR_013291
Homer	(Heinz et al., 2010)	http://homer.ucsd.edu/homer/; RRID: SCR_010881
ChromVAR	(Schep et al., 2017)	https://github.com/GreenleafLab/chromVAR
BWA algorithm	Broad Institute	https://arxiv.org/abs/1303.3997
GATK	(McKenna et al., 2010)	https://gatk.broadinstitute.org/; RRID: SCR_001876
Strelka2	(Kim et al., 2018)	https://github.com/Illumina/strelka
Manta	(Chen et al., 2016)	https://github.com/Illumina/manta
VarDict	(Lai et al., 2016)	https://github.com/AstraZeneca-NGS/VarDict
Ensembl VEP	(McLaren et al., 2016)	https://useast.ensembl.org/info/docs/tools/vep/index.html; RRID: SCR_007931

### Lead Contact and Materials Availability

Further information and requests for resources and reagents should be directed to and will be fulfilled by the Lead Contact, Claus Nerlov (claus.nerlov@imm.ox.ac.uk). The generation of the *Gata2* G320D mouse strain is described below and the mouse line is available upon request.

### Experimental Model and Subject Details

#### Animals

All mouse lines were maintained on a pure C57Bl/6J genetic background. All mice were bred and maintained in accordance with UK Home Office regulations. Experiments were conducted following ethical approval by the University of Oxford Medical Sciences Division Animal Welfare and Ethical Review Body under a project license form the UK Home Office (license number 30/3359).

Knock-in mice expressing the *Gata2* G320D (*Gata2*^*D/+*^) allele were generated by Cyagen Biosciences Inc, California, USA on a C57Bl6/J background. The G320D mutation was introduced into exon four by site-directed mutagenesis. The target vector contained a Neomycin resistance (Neo) cassette flanked by Frt sites and a thymidine kinase cassette used for negative selection of directly integrated vectors (Figure S1A). After homologous recombination in ES cells and germ line transmission of the correctly targeted allele, the Neo cassette was removed by Flp-mediated recombination.

*Gata2*^*D/+*^ mice were combined with knock-in mice containing a C-terminal *Cebpa* mutation (lysine insertion after C/EBPα amino acid 313; K313KK mice or K allele (Bereshchenko et al., 2009)). *Gata2*^D/+^;*Cebpa*^K/+^ mice were then time-mated to knock-in mice carrying a STOP codon in the p42-specific N-terminal part of C/EBPα (L-allele; (Kirstetter et al., 2008)) to produce single (*Gata2*^D/+^), double (*Cebpa*^K/L^) and triple transgenic mutant FL cells (*Cebpa*^K/L^;*Gata2*^D/+^), as well as WT control FL cells. *Gata2*^D/+^ mice were bred to homozygosity and primary *Gata2*^D/+^ and *Gata2*^D/D^ mice were analyzed between 4-5 weeks of age. Genotyping was performed using primers in Table S7.

#### Human BM Samples

AEL samples were obtained from MDSBio and were consented for research purposes. Sample OX1164 was a 48 year old female with add(3q), add(5q) cytogenetics. Sample AYL050 was a 47 year old female and was negative for *NPM1*, *FLT3* ITD and *FLT3* D835 mutations. Sample MKH048 was a 69 year old male and sample STB115 was a 73 year old male. There is no mutational or cytogenetic data for samples MKH048 and STB115. All samples were analyzed using flow cytometry. Samples OX1164 and AYL050 were subjected to single cell RNA sequencing. Normal adult human BM was obtained from AllCells (AllCells, California, USA).

#### Cell Lines

HEK293T/17 cells (ATCC, Virginia, USA) used for barcoding library virus product were cultured in DMEM (Thermo Fisher Scientific, Massachusetts, USA) with 10% FSC (Thermo Fisher Scientific), NEAA (Thermo Fisher Scientific) and 2 mM L-glutamine (Thermo Fisher Scientific) and incubated in 37°C, in 5% CO_2_, with ≥95% humidity. Virus was prepared when the cells were within six passages after they were obtained from ATCC, without further cell line authentication.

### Method Details

#### Competitive Transplantation

CD45.1/CD45.2*-Gata1*-EGFP adult mice (8-13 weeks old) were utilized as recipients (Drissen et al., 2016). Competitive transplantations were performed by using 2.5^∗^10^5^ CD45.2 FL cells and 2.5^∗^10^5^ CD45.1/CD45.2-*Gata1*-EGFP BM competitor cells into lethally irradiated recipients (two times 500 rads). For pre-leukemia analysis mice were culled at 6 weeks post-transplantation. For leukemia analysis mice were monitored up to 52 weeks post-transplantation. Mice were culled earlier if mice became hunched with pale paws, if PB WBC count was ≥15^∗^10^9^/L, or if RBC count was ≤7^∗^10^12^/L.

For secondary transplants lethally irradiated CD45.1/CD45.2*-Gata1*-EGFP adult mice (8-13 weeks old) were utilized as recipients. Bulk secondary transplants were performed by transplanting 5-7.5^∗^10^5^ BM cells with 2.5^∗^10^5^ CD45.1/CD45.2-*Gata1*-EGFP BM cells for radioprotection into CD45.1/CD45.2*-Gata1*-EGFP lethally irradiated recipients. For secondary transplants using sorted cell populations, all cells that were collected from the sort were split into two recipients with 2.5^∗^10^5^ CD45.1/CD45.2-*Gata1*-EGFP BM cells for radioprotection into CD45.1/CD45.2*-Gata1*-EGFP lethally irradiated recipients. Secondary transplants were also performed by sorting 50, 200 and 500 L-NMP, L-EB (defined as LKCD45^–^), and L-EoMPs from a KLG-E mouse with 2.5^∗^10^5^ CD45.1 or CD45.1/2 BM cells for radioprotection into CD45.1 or CD45.1/2 lethally irradiated recipients.

For comparing WT, *Gata2*^D/+^ and *Gata2*^D/D^ BM cell (CD45.2 allotype) competitive reconstitution adult mice (7-12 weeks old; CD45.1/2 allotype) were utilized as recipients. Competitive transplantations were performed using 5^∗^10^5^ CD45.2 BM donor cells and 5^∗^10^5^ CD45.1 WT BM competitor cells into lethally irradiated recipients (two times 500 rads). Bulk secondary transplants were performed by transplanting 10^∗^10^6^ BM cells, from primary transplanted mice 17-18 weeks post-transplantation, into lethally irradiated CD45.1/2 recipients.

#### Flow Cytometry

Details of murine antibodies and viability dyes used for each staining panel are shown in Table S8. All antibodies were used at pre-determined optimal concentrations. Hematopoietic stem and progenitor cells, myelo-erythroid progenitors, leukemic myeloid cells, erythroblast stages, platelets and erythrocytes were analyzed as previously described (Bereshchenko et al., 2009, Carrelha et al., 2018, Drissen et al., 2016, Drissen et al., 2019, Pronk et al., 2007, Socolovsky et al., 2001). In staining where anti-FcγRII/III antibody was not included, cells were pre-incubated with Fc-block. Gates were set using a combination of fluorescence minus one controls and populations known to be negative for the antigen. Cell acquisition and analysis were performed on a BD LSRFortessa (BD Biosciences, California, USA) using BD FACSDiva™ software (BD Biosciences). Cell sorting was performed on a BD FACSAriaII cell sorter (BD Biosciences). Analysis was performed using Flowjo software version 10.0.8 (Flowjo LLC, Oregon, USA).

#### RNA Sequencing Library Preparation

100 MB (defined as 7AAD^–^cKit^lo^Mac-1^+^CD45.2^+^), EB (defined as 7AAD^–^CD45.1^–^EGFP^–^CD71^hi^Ter119^lo^c-Kit^+^) and NMP (defined as LKFcγRII/III^+^CD45.2^+^) per biological replicate, from pre-leukemic and leukemic stages, were sorted into 4 μl of lysis buffer containing; 0.2% Triton X-100 (Sigma-Aldrich, Missouri, USA), 2.5 μM OligodT (Biomers, Ulm, Germany), 2.5 mM dNTPs (Thermo Fisher Scientific), RNase Inhibitor 20 U (Takara Bio USA, Inc, California, USA) and ERCC spike-in 1:4^∗^10^6^ (Thermo Fisher Scientific). cDNA synthesis and PCR amplification were performed based on the published Smart-seq2 protocol (Picelli et al., 2014) with some modifications. SMARTScribe RT enzyme (Takara Bio USA, Inc) was used in the RT mix (50 U) and SeqAMP enzyme (Takara Bio USA, 50 U) was used for the PCR step for 18 cycles. cDNA traces were bead-purified using Ampure XP beads (Beckman Coulter, California, USA). cDNA was evaluated using a high sensitivity NGS fragment analysis kit (Advanced Analytical, Milton Keynes, UK) on a Fragment Analyzer. cDNA was quantified using PicoGreen (Thermo Fisher Scientific). Normalized cDNA traces were used for library preparation using a miniaturized version of the Nextera XT Kit (Illumina, California, USA). After tagmentation and 12 cycles of barcoding PCR, tagmented libraries were purified using AmpureXP beads, evaluated using a high sensitivity DNA kit on an Agilent 2100 Bioanalyzer (Agilent, California, USA) and quantified using a Qubit (Invitrogen, California, USA). Finally, libraries were pooled and sequenced on four lanes on a NextSeq 500 (Illumina), using 75 bp single-end reads.

#### Cell Culture

100 *Cebpa*^K/L^;*Gata2*^D/+^ or 300 WT CD45.2 LKFcγRII/III^+^ BM cells were sorted from mice transplanted with 2.5^∗^10^5^ CD45.2 FL cells and 2.5^∗^10^5^ CD45.1/CD45.2 BM competitor cells 6 weeks post-transplantation. Cells were seeded into 1 ml of methylcellulose medium (Methocult, M3434, STEMCELL Technologies) and incubated in 37°C, in 5% CO_2_, with ≥95% humidity. After 8 days colonies (≥30 cells) were counted and colonies were picked by taking 1 μl of cells from the colony and re-suspending in a well of a 96 well plate containing 200 μl PBS + 5% FCS. Cell suspension was then split into two separate plates. Both plates were spun down at 500 g for 5 mins at 4°C. Supernatant was then removed. One plate was re-suspended in 15 μl of lysis buffer containing 14.85 μl of CellDirect 2x reaction mix (Thermo Fisher Scientific) and 0.15 μl of SUPERase-In RNase Inhibitor (Thermo Fisher Scientific), then flash frozen on dry ice and stored at –80°C for multiplex qRT-PCR analysis. The second plate was re-suspended in 20 μl of PBS + 20% FCS to be used to make a cytospin.

#### Multiplex qRT-PCR Analysis

Multiplex quantitative real-time PCR was performed on single cells, 50 cells, or picked colonies from methylcellulose cultures. CellDirect One-Step qPT-PCR kit (Thermo Fisher Scientific) was used according to manufacturer’s protocol for preparation and amplification of cDNA. The BioMark 192.24 Dynamic Array platform (Fluidigm, California, USA) and Taqman assays (Thermo Fisher Scientific) were used to perform the multiplex qRT-PCR according to the manufacturer’s instructions (Table S7).

#### Morphology and Cell Counts

Blood smears were made using 3.5 μl of blood. 10^∗^10^4^ BM or spleen cells were used to make cytospins. Air-dried cytopsins and blood smears slides were stained with May-Grünwald (Sigma-Aldrich) and Giemsa (Sigma-Aldrich) reagents. WBC, RBC and platelet parameters from the PB were measured using a Sysmex KX-21N (Sysmex, Milton Keynes, UK).

#### *In Vivo* Barcoding

5^∗^10^5^ *Cebpa*^K/L^;*Gata2*^D/+^ leukemic BM cells were co-transplanted with 2.5^∗^10^5^ CD45.1 BM cells into lethally irradiated CD45.1 recipients. Four weeks post-transplantation mice were culled and 1^∗^10^5^ CD45.2^+^LKFcγRII/III^+^CD55^–^ BM cells were sorted and cultured in IMDM with 0.05% BSA (Thermo Fisher Scientific), penicillin/streptomycin (Invitrogen), 0.1 μM β-mercaptoethanol (Sigma-Aldrich), and 4 μg/ml hexadimethrine bromide (Sigma-Aldrich), supplemented with 50 ng/ml mSCF (Peprotech, New Jersey, USA), 10 ng/ml hIL-6 (Peprotech), and 10 ng/ml mIL-3 (Peprotech). To generate the pEGZ2 lentiviral barcoding library (Belderbos et al., 2017) (total 725 different barcodes) HEK293T cells (ATCC, Virginia, USA) were transfected with the pGIPZ-based library, pMD2.G and psPAX2 plasmids using PEI Pro (Polyplus Transfection, New York, USA). Harvests were collected 48 and 72 h post transfection, combined and concentrated by ultracentrifugation (2 h at 98,000 g, 4°C). Cells were transduced with barcoding library at an MOI of 10, defined as the titre on HEK293T cells divided by the number of L-NMPs. This generated an L-NMP infection rate of ca. 15%. Cells were incubated at 37°C, 5% CO_2_, for 8 h. Cells were then co-transplanted with 2.5^∗^10^5^ WT CD45.1 BM cells into CD45.1 lethally irradiated recipients. Three weeks post-transplantation mice were culled. Transduced leukemic NMPs (CD45.2^+^GFP^+^LKFcγRII/III^+^CD55^–^) from the BM, transduced leukemic erythroblasts (CD45.1^–^GFP^+^CD71^hi^Ter119^lo^) and transduced leukemic myeloblasts (CD45.2^+^GFP^+^c-Kit^lo^Mac1^+^) were sorted from BM and spleen. Leukemic erythroblasts and myeloblasts were pooled separately, and DNA was extracted from cell pellets using a QIAamp DNA micro kit (Qiagen, Maryland, USA). DNA was quantified using a Qubit (Invitrogen). Barcode sequences were amplified with primers designed around the barcoding region with Nextera XT compatible overhangs allowing for indexing and a stagger sequence in the forward primers between Nextera XT compatible overhangs and forward sequence to defer cluster calling when sequencing (Krueger et al., 2011) (Table S7). PCR products were bead-purified using Ampure XP beads. PCR products were then evaluated using a high sensitivity DNA kit on an Agilent 2100 Bioanalyzer, and quantified using a Qubit. 15 ng of PCR products were used for library preparation using a Nextera XT kit. Libraries were purified using AmpureXP beads, evaluated using a high sensitivity DNA kit on an Agilent 2100 Bioanalyzer, and quantified using a Qubit. Finally, libraries were pooled and sequenced on a MiSeq (Illumina), using 150 bp paired-end reads.

#### Single Cell 10x Chromium Library Preparation

8700 Lin^–^CD71^+^CD235a^+^ and 8700 Lin^–^CD71^–^CD235a^–^ single cells were sorted from two human AEL samples. Libraries were preparing using the chromium single cell 3’ reagent kits v2 (10x Genomics, California USA) according to manufacturer’s protocol.

#### ATAC Sequencing Library Preparation

500 L-NMPs were sorted into lysis buffer containing TD tagmentation buffer (Illumina), 1% digitonin (Promega, Wisconsin USA), 10% Tween 20 (Sigma-Aldrich) and PBS (Thermo Fisher Scientific). After cells were sorted into the lysis buffer the Tn5 transposase was then added and immediately incubated at 37°C for 30 mins with an agitation at 300 rpm. Samples were then purified using a Qiagen MinElute Kit (Qiagen). Samples were then PCR amplified and indexed using NEBNext High-Fidelity 2x PCR master mix (NEB, Massachusetts USA) and P7 and P5 primers containing Nextera adaptor sequences (Table S7). PCR products were then purified using Ampure XP beads and evaluated using a High Sensitivity D1000 Screen Tape (Agilent) on a TapeStation (Agilent). Samples were quantified using an NEBNext Library Quant Kit for Illumina (NEB). Finally, libraries were pooled and sequenced on a NextSeq (Illumina), using 40 bp paired-end reads; 40 cycles R1 and 40 cycles R2.

#### Whole Exome Sequencing Library Preparation

DNA was extracted from frozen cell pellets using a QIAamp DNA minikit (Qiagen) and DNA quantified using a Qubit. Exomes were captured using a Aglient SureSelect Mouse All Exon Kit (Agilent, California, USA), libraries were sequenced on a HiSeq (Illumina), using 150 bp paired-end reads.

### Quantification and Statistical Analysis

#### Flow Cytometry

For significance testing of blood analysis the D’Agostino & Pearson normality test was first used to determine if data fell into a normal distribution. If data did not have a normal distribution then a multiple comparison Kruskal-Wallis test was performed. If the data had a normal distribution then a multiple comparison ANOVA was performed.

#### RNA Sequencing Analysis

Following quality control analysis with the fastQC package (http://www.bioinformatics.babraham.ac.uk/projects/fastqc), reads were aligned using STAR (Dobin et al., 2013) against the mm10 mouse reference genome. Gene expression levels were quantified as read counts using the featureCounts function from the Subread package (Liao et al., 2013) with default parameters. The read counts were used for the identification of global differential gene expression between specified populations and/or genotypes using the DESeq2 package (Love et al., 2014). Reads per kilobase of transcript per million (RPKM) values were then calculated. Genes were considered differentially expressed between populations and/or genotypes if they had an adjusted p value of less than 0.05. The pheatmap function was used to generate a heatmap, and prcomp function was used to generate a principal component analysis, in R statistical programming environment (www.r-project.org). Gene-set enrichment analysis (GSEA) was performed using GSEA software (Mootha et al., 2003, Subramanian et al., 2005) using previously described preGM, MegE, preCFU-E (Bereshchenko et al., 2009, Mancini et al., 2012) and neutrophil gene sets (de Graaf et al., 2016).

#### Multiplex qRT-PCR Analysis

Ct values were generated using the BioMark Real-Time PCR analysis software (Fluidigm). Each amplification curve for each gene and each cell was visually inspected on the BioMark Real-Time PCR analysis software. Any outliers that were not automatically detected from the software were manually changed to fail. Data analysis was then performed in R statistical programming environment. Ct values of all assays marked as ‘Fail’ were set as undetected (Ct = 999). A histogram was generated using Excel (Microsoft, Washington, USA) to analyze the Ct values for the housekeeping genes. Cut-offs’ for the housekeeping genes Ct values were set in accordance to the histogram analysis. Cells that had a housekeeping gene Ct value that did not meet the cut-off, or were undetected, were removed from analysis. Ct values were then normalized to the housekeeping gene. If more than one housekeeping gene was used in the assay then a mean was calculated for the housekeeping genes. Ct values were normalized to the mean of the housekeeping genes. 2^–(Normalized Ct)^ was then used for analysis. 2^–(Normalized Ct)^ values for each gene were visually inspected and outliers removed. Differential gene expression statistical significance between genotypes was performed using the Wilcoxon signed-rank test. Differential gene expression frequency statistical significance between genotypes was performed using a Fisher’s exact test. P value from the two tests were combined using Fisher’s method. Pheatmap function was used to generate a heatmap. The sum of myeloid and E/Mk genes detected in each cell was used to generate scatterplots using ggplot2 function in R statistical programming environment.

#### Barcode Analysis

Raw fastq sequencing data files were demultiplexed using Illumina indices and analyzed using a custom-written script in R statistical programming environment. All reads were searched for sequencing matching the following barcode region: GGNNNACNNNGTNNNTANNNCANNNTGNNN. Barcodes with exact matches with a minimum representation of one read in the sample with lowest sequencing depth were included in subsequent analysis. Venn-diagrams were generated using the VennDiagram R package. P values were calculated using the hypergeometric test for 2-way overlap (probability of achieving the obtained overlap of L-EB and L-MB barcodes by chance from a pool of 725 barcodes) and random draw simulation (10,000,000 iterations) for 3-way overlap (probability of the observed number of barcodes being present in all three populations from the pool of identified barcodes).

#### Gene Signatures

Leukemic stem cell signature was generated using the upregulated genes identified in leukemic stem cells from previously published data (Ng et al., 2016). Erythroblast gene signature was generated by selecting the top 200 upregulated genes (adjusted p value<0.05) from erythroblasts compared to long-term HSCs from previously published data (de Graaf et al., 2016). Myeloblast gene signature was generated by selecting the overlapping upregulated genes (adjusted p value<0.05, fold change>2) from KLG-E L-MBs compared to KLG-E L-EBs and KLG-E L-MBs compared to KLG-E L-NMPs. Human M6 AEL was generated by selecting the top 200 up-regulated genes from AEL (FAB: M6) compared to all other AML samples from previously published data (GEO: GSE14468). BiomaRt was used to interconvert human and mouse gene names in R. Signatures are available upon request.

#### Single Cell 10x Chromium Analysis

Gene count matrix for each sample was generated using Cell Ranger software (10x Genomics). Sample integration, cluster and gene expression analysis were performed using Seurat (Butler et al., 2018). tSNE of leukemic stem cell, erythroblast and myeloblast gene signatures were generated using the AddModuleScore function in Seurat with default settings.

#### ATAC Sequencing Analysis

Sequences were trimmed using Trim Galore (https://github.com/FelixKrueger/TrimGalore) and mapped to the mm10 murine reference genome using Bowtie2 (Langmead and Salzberg, 2012). SAMtools was then used to convert sam files to bam files (Li et al., 2009). Duplicates were then removed using MarkDuplicates function from the Picard tools package (http://broadinstitute.github.io/picard/). Bam files were subsampled and merged using SAMtools. Peaks were called using MACS2 with default parameters (Zhang et al., 2008). Regions of chromatin accessibility was quantified as peak counts using the featureCounts function from the Subread package using default parameters. Peaks were annotated using Homer (Heinz et al., 2010). Differential peak analysis was performed using the DESeq2 package. Genes with differentially accessible promoters (p value<0.05; log2 fold change>1.5) were identified by integrating peaks within 1kb of the transcription start site, and were used to calculate promoter accessibility correlation. Motif accessibility analysis was performed with ChromVAR using the mouse_pwms_v1 TF motif collection (Schep et al., 2017). Sample correlation was calculated using the getSampleCorrelation function. Variance of motif accessibility across samples was calculated using the deviationsScore function and the average deviation score calculated for preleukemia genotypes and leukemia phenotypes. Correlation between deviation scores and promoter accessibilities was calculated using linear modelling in R after filtering for significance (p value<0.005 for motifs, p value<0.05 and Log2Fc>1.5 for promoters).

#### Mutational Analysis by Whole Exome Sequencing

Somatic variants were called using a custom pipeline. Pre-processing was performed according to GATK best practice. Read alignment to the mm10 reference genome was performed with the BWA algorithm (v0.7.17; https://arxiv.org/abs/1303.3997), with corrections with GATK4 BaseRecalibrator (v4.0.5.1) (McKenna et al., 2010) after removal of PCR duplicates with Picard MarkDuplicates (v2.18.7). Somatic variant detection was carried out using three variant callers: GATK4 Mutect2 (v4.0.5.1; t_lod>=3.5), Strelka2 (v.2.9.2 after running Manta v.1.3.2; EVS>=5 for SNVs) (Chen et al., 2016, Kim et al., 2018) and Vardict (v.2018.10.18) (Lai et al., 2016). Artefact variants due to DNA oxidation resulting in G to T transversion during library preparation were filtered out using GATK FilterByOrientationBias. Annotation was performed using Ensembl VEP (v.98) (McLaren et al., 2016). Somatic variants were defined as the overlap of at least two out of the three variant callers with VAF>5%, with a minimum of 10 reads and filtered to exclude non-coding and synonymous variants.

### Data and Code Availability

RNA-sequencing (GEO: GSE121492), ATAC-sequencing (GEO: GSE141812) and 10x RNA-sequencing (GEO: GSE142213) data have been deposited in GEO under the SuperSeries accession number: GEO: GSE141813. Previously published expression data used to create the human AEL M6 gene signature is available through GEO under GEO: GSE14468. The R code supporting the study is available from the Lead Contact on request.

## Supplemental Information

Document S1. Figures S1–S8 and Tables S1–S3, S7, and S8

Table S4. Differential Gene Expression between Pre-leukemic and Leukemic Erythroblasts and Myeloblasts, Related to Figure 4 and 5

Table S5. Exome Sequencing of KLG-M and KLG-E Leukemias, Related to Figure 6

Table S6. ATAC Sequencing Peak Intensities in NMP and L-NMP Samples, Related to Figure 6

Document S2. Article plus Supplemental Information
