# Supplementary material for: C/EBPα and GATA-2 Mutations Induce Bilineage Acute Erythroid Leukemia through Transformation of a Neomorphic Neutrophil-Erythroid Progenitor
Source: Cancer Cell. 2020 May 11;37(5):690–704.e8. doi: 10.1016/j.ccell.2020.03.022 (PMC7218711; doi:10.1016/j.ccell.2020.03.022)
Supplement: Document S1. Figures S1–S8 and Tables S1–S3, S7, and S8 [file mmc1.pdf]

Cancer Cell, Volume *37*

Supplemental Information

C/EBPα and GATA-2 Mutations Induce Bilineage Acute Erythroid Leukemia through Transformation of a Neomorphic Neutrophil-Erythroid Progenitor

Cristina Di Genua, Simona Valletta, Mario Buono, Bilyana Stoilova, Connor Sweeney, Alba Rodriguez-Meira, Amit Grover, Roy Drissen, Yiran Meng, Ryan Beveridge, Zahra Aboukhalil, Dimitris Karamitros, Mirjam E. Belderbos, Leonid Bystrykh, Supat Thongjuea, Paresh Vyas, and Claus Nerlov

Cancer Cell, Volume *37*

Supplemental Information

C/EBPα and GATA-2 Mutations Induce Bilineage Acute Erythroid Leukemia through Transformation of a Neomorphic Neutrophil-Erythroid Progenitor

Cristina Di Genua, Simona Valletta, Mario Buono, Bilyana Stoilova, Connor Sweeney, Alba Rodriguez-Meira, Amit Grover, Roy Drissen, Yiran Meng, Ryan Beveridge, Zahra Aboukhalil, Dimitris Karamitros, Mirjam E. Belderbos, Leonid Bystrykh, Supat Thongjuea, Paresh Vyas, and Claus Nerlov

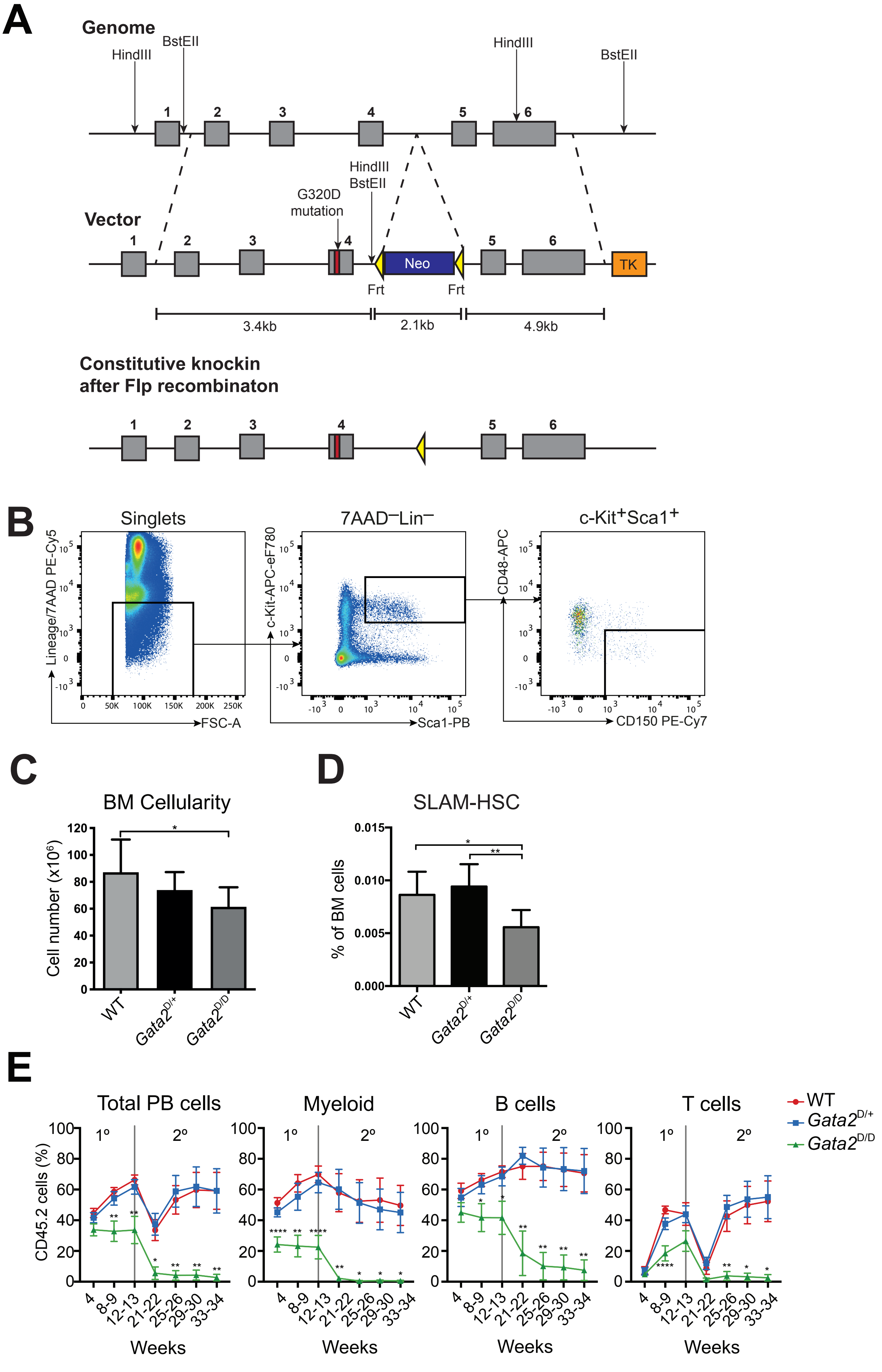
A
Genome
HindIII
BstEII
HindIII
BstEII
1
2
3
4
5
6
Vector
G320D
mutation
HindIII
BstEII
Neo
TK
Frt
Frt
3.4kb
2.1kb
4.9kb
Constitutive knockin
after Flp recombinaton
B
Singlets
7AAD⁻Lin⁻
c-Kit⁺Sca1⁺
Lineage/7AAD PE-Cy5
FSC-A
c-Kit-APC-eF780
Sca1-PB
CD48-APC
CD150 PE-Cy7
C
BM Cellularity
Cell number (x10⁶)
WT
Gata2D/+
Gata2D/D
D
SLAM-HSC
% of BM cells
E
Total PB cells
Myeloid
B cells
T cells
CD45.2 cells (%)
1º
2º
Weeks
WT
Gata2D/+
Gata2D/D

Figure S1

Figure S1, related to Figure 1. Generation of the *Gata2* G320D knock-in mutant model.

(A) Schematic of *Gata2* G320D mutant knock-in design. Closed grey boxes, exons; closed purple box, neomycin resistance cassette; triangles, FRT-sites; Closed orange box, thymidine kinase expression cassette. HindIII and BstEII indicate the restriction sites used for Southern blot analysis of targeted embryonic stem (ES) cell clones.

(B) Gating strategy for SLAM-HSCs defined as $Lin^{-}Sca\text{-}1^{+}c\text{-}Kit^{+}$ (LSK) $CD150^{+}CD48^{-}$.

(C) Total bone marrow (BM) cellularity was measured in mice of the indicated genotypes (n=8-12; 4 independent experiments). The results are presented as the mean ± SD. *p value<0.05

(D) SLAM-HSCs measured as shown in (B) as a percentage of the BM in mice from (C). The results are presented as the mean ± SD. Significance was determined using multiple-comparison ANOVA. *p value<0.05; **p value<0.01.

(E) Percentage reconstitution of CD45.2 cells in total peripheral blood (PB) cells, myeloid, B and T cell compartments (n=8-10/genotype, for primary transplants and n=6/genotype for secondary transplants; 2 independent experiments). Secondary transplant was performed by total BM transfer at week 12-13; the x-axis shows time elapsed from start of first transplantation. The results are presented as the mean ± SEM. Statistical significance (multiple comparison ANOVA) between WT and *Gata2*$^{D/D}$ are shown. *p value<0.05 **p value<0.01 ***p value<0.001 ****p value<0.0001.

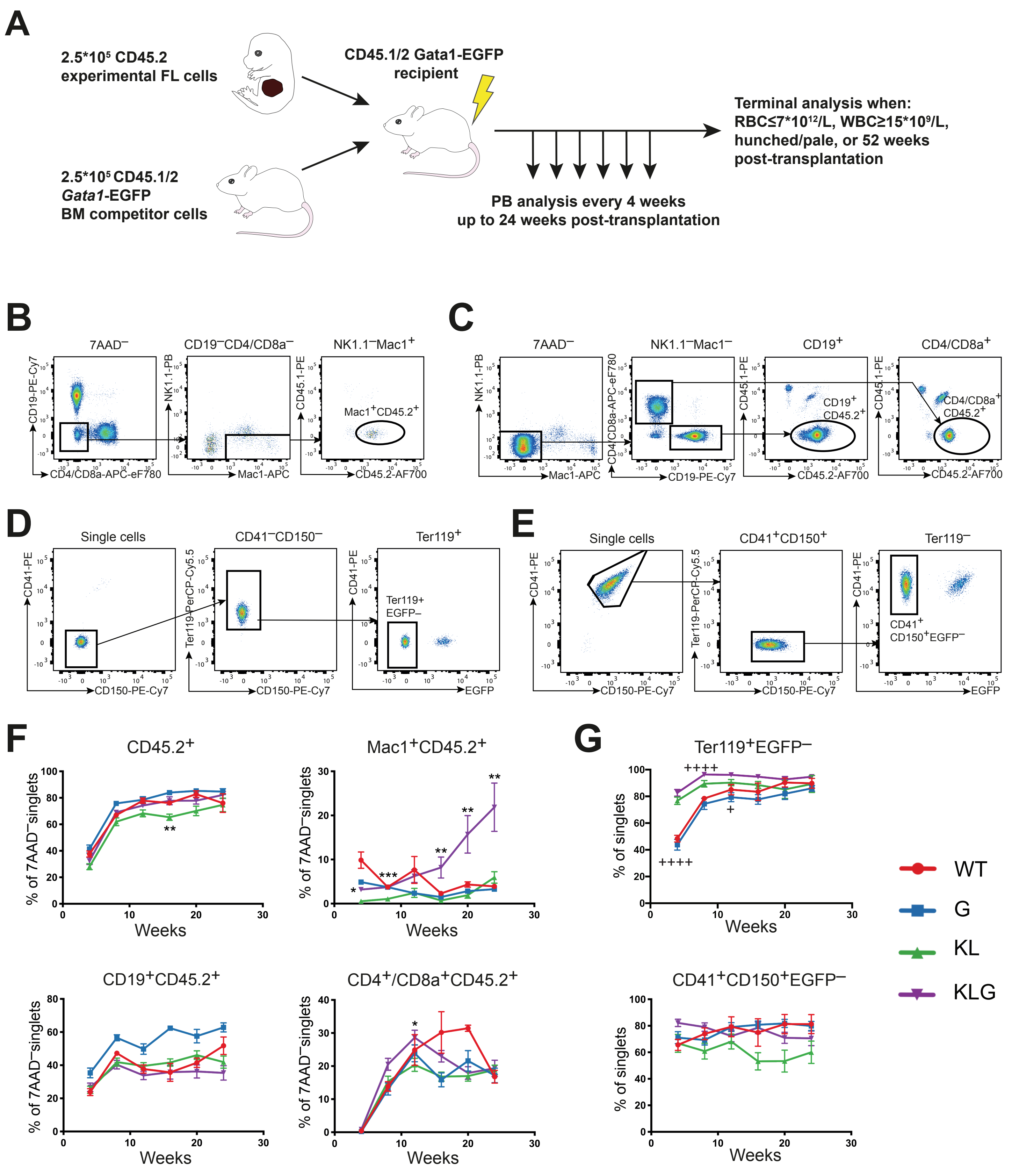
A
2.5*10⁵ CD45.2
experimental FL cells
2.5*10⁵ CD45.1/2
Gata1-EGFP
BM competitor cells
CD45.1/2 Gata1-EGFP
recipient
PB analysis every 4 weeks
up to 24 weeks post-transplantation
Terminal analysis when:
RBC≤7*10¹²/L, WBC≥15*10⁹/L,
hunched/pale, or 52 weeks
post-transplantation
B
7AAD−
CD19−CD4/CD8a−
NK1.1−Mac1+
Mac1+CD45.2+
CD19-PE-Cy7
CD4/CD8a-APC-eF780
NK1.1-PB
Mac1-APC
CD45.1-PE
CD45.2-AF700
C
7AAD−
NK1.1−Mac1−
CD19+
CD4/CD8a+
CD19+ CD45.2+
CD4/CD8a+ CD45.2+
CD19-PE-Cy7
D
Single cells
CD41−CD150−
Ter119+
Ter119+ EGFP−
CD41-PE
CD150-PE-Cy7
Ter119-PerCP-Cy5.5
EGFP
E
CD41+CD150+
Ter119−
CD41+ CD150+EGFP−
F
CD45.2+
Mac1+CD45.2+
CD19+CD45.2+
CD4+/CD8a+CD45.2+
% of 7AAD−singlets
Weeks
G
Ter119+EGFP−
CD41+CD150+EGFP−
% of singlets
WT
G
KL
KLG

Figure S2

Figure S2, related to Figure 1. Biallelic *Cebpa* combined with *Gata2* ZnF1 mutation causes an increase in myeloid reconstitution over time in the PB.

(A) Schematic of *in vivo* competitive transplantation experiment.

(B-E) Gating strategy for myeloid cells (B), lymphoid cells (C), erythrocytes (D) and platelets (E).

(F) PB reconstitution over time in donor derived CD45.2 cells. Total CD45.2^{+} cells (CD45.2^{+}), myeloid cells (Mac1^{+}CD45.2^{+}), B cells (CD19^{+}CD45.2^{+}), and T cells (CD4^{+}/CD8^{+}CD45.2^{+}) are shown as a percentage of 7AAD^{-} singlets in WT (n=9), G (n=10), KL (n=15) and KLG (n=18) mice from 4 independent experiments.

(G) PB reconstitution over time in donor derived EGFP^{-} erythrocytes (Ter119^{+}) and platelets (CD41^{+}CD150^{+}) as a percentage of singlets in WT (n=9), G (n=7), KL (n=11) KLG (n=14) mice from 3 independent experiments. The results are presented as the mean ± SEM. The results were analyzed using a multiple comparison ANOVA. Statistical significance of KLG vs KL (*p value<0.05; **p value<0.01, ***p value<0.001) and KLG vs. WT comparison (+p value<0.05; ++++p value<0.0001).

Table S1, Related to Figure 1. Leukemia characteristics.

Genotype	Behavior	WBC (x10^{9}/L)	RBC (x10^{12}/L)	Latency (weeks)
WT	NA	13.5	8.2	51
WT	NA	9	6	51
WT	NA	10.5	7.7	53
WT	NA	13.5	8.1	53
WT	NA	12.5	7.82	53
WT	NA	11.5	8.15	53
G	NA	6.5	7.75	52
G	NA	8	7.55	52
G	NA	24.5	8.4	52
G	NA	12.5	8.3	52
G	NA	16	7.35	52
G	NA	8.5	6.45	51
G	NA	13.5	9	51
G	NA	25	7	51
G	NA	15	7	51
KL	Hunched, pale, inactive	24.5	4	47
KL	Lost weight	174	2.65	35
KL	Hunched, pale	112.5	1.45	46
KL	Pale	28.3	1.7	36
KL	Hunched, inactive	42.5	7.3	29
KL	Hunched, pale	238.5	2	49
KL	Died	NA	NA	45
KL	Hunched, pale, inactive	68.5	2.4	33
KL	NA	14	1.45	53
KL	NA	10	5.55	53
KL	Pale	23	0.9	34
KL	NA	NA	NA	32
KL	Hunched, pale	17	1.1	38
KLG-M	Hunched	135	3.05	45
KLG-M	Hunched, pale, inactive	152.5	1.1	37
KLG-M	NA	47.4	6.99	24
KLG-M	Pale	40	2.6	30
KLG-M	NA	15.2	7.37	24
KLG-M	Hunched, pale	97.5	2.3	37

KLG-M	Hunched, pale	74	2.9	40
KLG-M	Hunched, pale	21	2	40
KLG-E	Hunched, pale, inactive	83.4	1.1	22
KLG-E	Hunched	371.5	3.8	29
KLG-E	Pale	23	0.95	25
KLG-E	Hunched, inactive, lost weight	499.2	4.12	34
KLG-E	Hunched, pale	173.5	0.65	22
KLG-U	Hunched, pale	200.2	1.55	31
KLG-U	Found dead	NA	NA	38
KLG-U	Hunched, pale	173.5	1.1	37
KLG-U	Hunched, pale	38	1.45	32
KLG-U	Hunched, pale	195	2.65	35
KLG-U	Hunched, pale	29.5	1.95	35
KLG-U	Hunched, pale	NA	NA	31
KLG-U	Hunched, pale	35.5	1.65	33
KLG-U	NA	NA	NA	32
KLG-U	NA	6	6.9	57

Appearance and PB parameters observed when mice were culled for terminal analysis. WBC: white blood cells; RBC: red blood cells; WT, wild type; G, *Gata2*$^{D/+}$ genotype; KL, *Cebpa*$^{K/L}$ genotype; KLG *Cebpa*$^{K/L}$*Gata2*$^{D/+}$ genotype; KLG-M, KLG myeloid leukemia; KLG-E, KLG erythroleukemia; KLG-U, KLG mice with an undetermined phenotype; NA, not applicable.

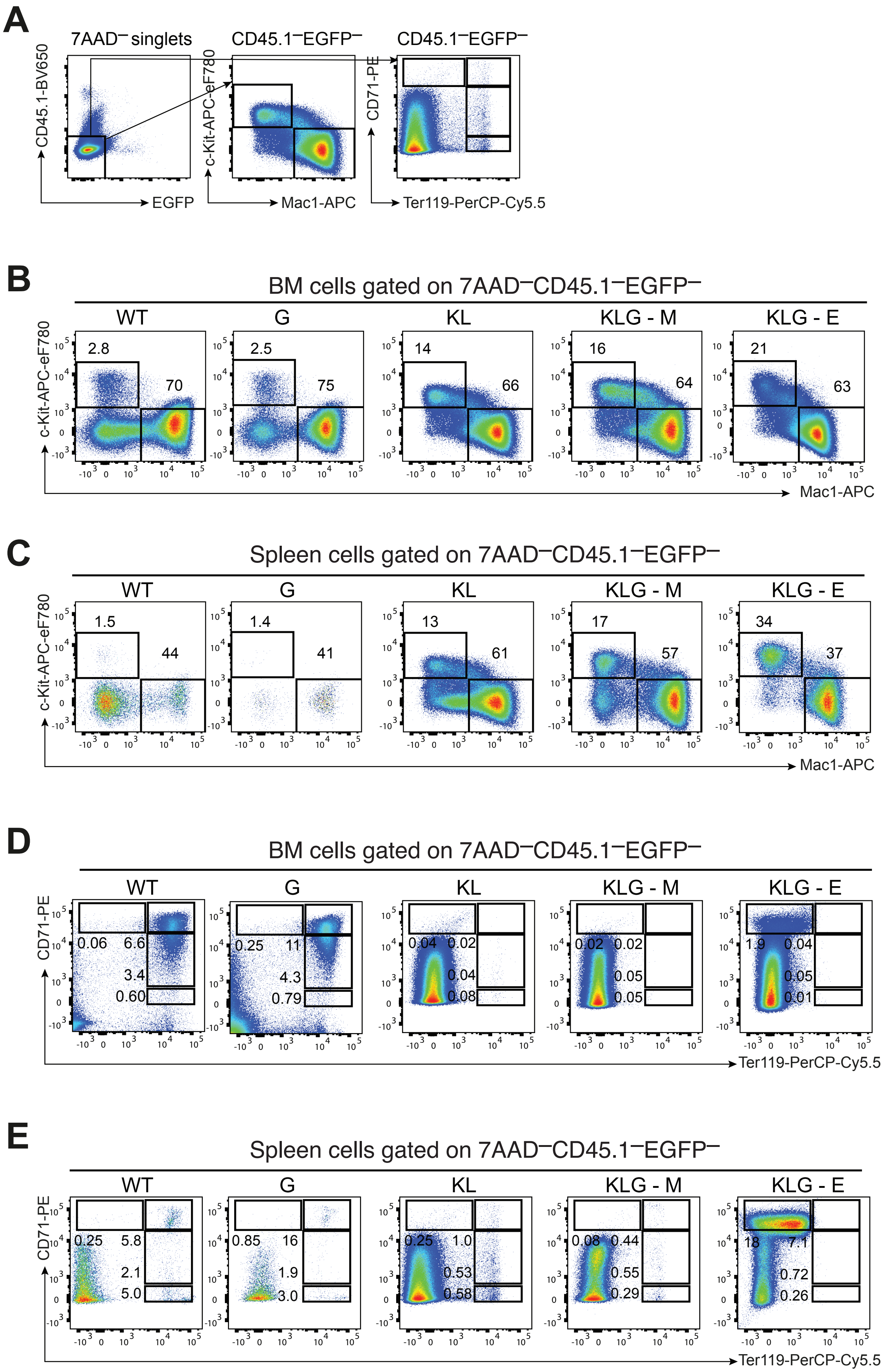
A
7AAD⁻ singlets
CD45.1⁻EGFP⁻
CD45.1⁻EGFP⁻
CD45.1-BV650
EGFP
c-Kit-APC-eF780
Mac1-APC
CD71-PE
Ter119-PerCP-Cy5.5
B
BM cells gated on 7AAD⁻CD45.1⁻EGFP⁻
WT
G
KL
KLG - M
KLG - E
c-Kit-APC-eF780
2.8
70
2.5
75
14
66
16
64
21
63
Mac1-APC
C
Spleen cells gated on 7AAD⁻CD45.1⁻EGFP⁻
WT
G
KL
KLG - M
KLG - E
c-Kit-APC-eF780
1.5
44
1.4
41
13
61
17
57
34
37
Mac1-APC
D
BM cells gated on 7AAD⁻CD45.1⁻EGFP⁻
WT
G
KL
KLG - M
KLG - E
CD71-PE
0.06
6.6
3.4
0.60
0.25
11
4.3
0.79
0.04
0.02
0.04
0.08
0.02
0.02
0.05
0.05
1.9
0.04
0.05
0.01
Ter119-PerCP-Cy5.5
E
Spleen cells gated on 7AAD⁻CD45.1⁻EGFP⁻
WT
G
KL
KLG - M
KLG - E
CD71-PE
0.25
5.8
2.1
5.0
0.85
16
1.9
3.0
0.25
1.0
0.53
0.58
0.08
0.44
0.55
0.29
18
7.1
0.72
0.26
Ter119-PerCP-Cy5.5

Figure S3

Figure S3, related to Figure 2. KLG-E mice contain erythroblasts in the BM and spleen.

(A) Gating strategy analysis of erythroid and myeloid lineage cells. CD45.1^{+} and EGFP^{+} cells are gated out to remove all competitor and recipient cells, including erythroid lineage competitor and recipient cells that are EGFP^{+}. Remaining cells are donor-derived cells.

(B) Representative flow cytometry analysis of experimental myeloid cells in the BM as a percentage of BM 7AAD^{-}CD45.1^{-}EGFP^{-} cells from terminal analysis of mice as described in Figure 2A with the indicated genotypes and phenotypes.

(C) Representative flow cytometry analysis of experimental myeloid cells in the spleen as a percentage of spleen 7AAD^{-}CD45.1^{-}EGFP^{-} cells as in (B).

(D) Representative flow cytometry analysis of experimental stage I-IV erythroblast cells in the BM as a percentage of BM 7AAD^{-}CD45.1^{-}EGFP^{-} cells from terminal analysis of mice as described in Figure 2B with the indicated genotypes and phenotypes.

(E) Representative flow cytometry analysis of experimental stage I-IV erythroblast cells in the spleen as a percentage of spleen 7AAD^{-}CD45.1^{-}EGFP^{-} cells as in (D).

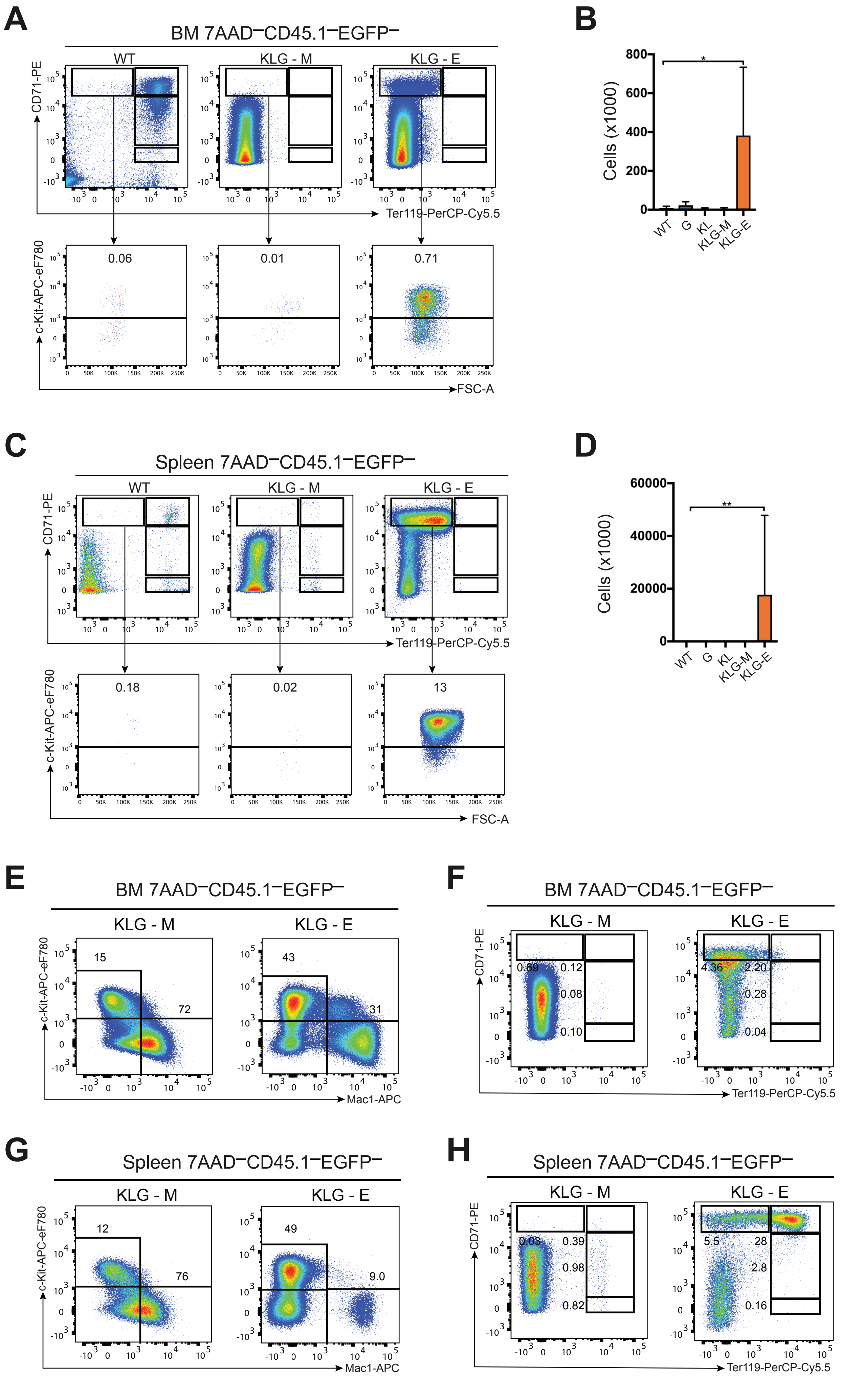

A
BM 7AAD⁻CD45.1⁻EGFP⁻
WT
KLG - M
KLG - E
CD71-PE
Ter119-PerCP-Cy5.5
0.06
0.01
0.71
c-Kit-APC-eF780
FSC-A
B
Cells (x1000)
WT
G
KL
KLG-M
KLG-E
C
Spleen 7AAD⁻CD45.1⁻EGFP⁻
0.18
0.02
13
D
E
BM 7AAD⁻CD45.1⁻EGFP⁻
15
72
43
31
Mac1-APC
F
0.69
0.12
0.08
0.10
4.36
2.20
0.28
0.04
G
Spleen 7AAD⁻CD45.1⁻EGFP⁻
12
76
49
9.0
H
0.03
0.39
0.98
0.82
5.5
28
2.8
0.16

Figure S4

Figure S4, related to Figure 2. Bilineage erythroid leukemia is transplantable.

(A) Representative flow cytometry analysis of c-Kit^{+} stage I pro-erythroblasts as a percentage of 7AAD^{-} CD45.1^{-}EGFP^{-} in BM in mice of the indicated transplant genotypes at terminal analysis as described in Figure 2B.

(B) Absolute number of c-Kit^{+} stage I pro-erythroblasts in BM of mice with the indicated transplant genotypes at terminal analysis from (A). Values are mean±SD. P values were calculated using the Mann-Whitney U-test. *p value<0.05.

(C) Representative flow cytometry analysis of c-Kit^{+} stage I pro-erythroblasts as a percentage of 7AAD^{-} CD45.1^{-}EGFP^{-} in spleen in mice of the indicated transplant genotypes at terminal analysis as described in Figure 2B.

(D) Absolute number of c-Kit^{+} stage I pro-erythroblasts in spleen of mice with the indicated transplant genotypes at terminal analysis from (C). Values are mean±SD. P values were calculated using the Mann-Whitney U-test. **p value<0.01.

(E) Representative flow cytometry analysis of c-Kit^{lo}Mac1^{+} and c-Kit^{+}Mac1^{lo} cells as a percentage of 7AAD^{-}CD45.1^{-}EGFP^{-} cells in the BM of mice transplanted with bulk BM cells from KLG-M and KLG-E leukemic mice as described in Figure 2C.

(F) Representative flow cytometry analysis of stage I-IV erythroblast cells as a percentage of 7AAD^{-} CD45.1^{-}EGFP^{-} cells in the BM of mice transplanted with bulk BM cells from KLG-M and KLG-E leukemic mice as described in Figure 2D.

(G) Representative flow cytometry analysis of c-Kit^{lo}Mac1^{+} and c-Kit^{+}Mac1^{lo} cells as a percentage of 7AAD^{-}CD45.1^{-}EGFP^{-} cells in the spleen of mice transplanted with bulk BM cells from KLG-M and KLG-E leukemic mice as described in Figure 2C.

(H) Representative flow cytometry analysis of stage I-IV erythroblast cells as a percentage of 7AAD^{-} CD45.1^{-}EGFP^{-} cells in the spleen of mice transplanted with bulk BM cells from KLG-M and KLG-E leukemic mice as described in Figure 2D.

Table S2, Related to Figure 2 and 3. Bulk and sorted populations tested for leukemic initiating potential

Sample No.	Phenotype	Population	Cell number	Leukemia (Y/N)	Latency (weeks)	Average latency (weeks)
97	KLG-M	Bulk	750000	Y	8	8
99	KLG-M	Bulk	750000	Y	8	
136	KLG-M	Bulk	500000	Y	4	
137	KLG-M	Bulk	500000	Y	4	
138	KLG-M	Bulk	500000	Y	17	
100	KLG-E	Bulk	750000	Y	6	5
101	KLG-E	Bulk	750000	Y	8	
102	KLG-E	Bulk	750000	Y	7	
121	KLG-E	Bulk	500000	Y	5	
122	KLG-E	Bulk	500000	Y	4	
123	KLG-E	Bulk	500000	Y	4	
139	KLG-E	Bulk	500000	Y	3	
140	KLG-E	Bulk	500000	Y	3	
141	KLG-E	Bulk	500000	Y	3	
107	KLG-M	L-NMP	2243	Y	9	8
108	KLG-M	L-NMP	2243	Y	9	
149	KLG-M	L-NMP	8934	Y	8	
151	KLG-M	L-NMP	8934	Y	8	
105	KLG-E	L-NMP	8509	Y	6	5
106	KLG-E	L-NMP	8509	Y	6	
126	KLG-E	L-NMP	1345	N	NA	
127	KLG-E	L-NMP	1345	Y	5	
132	KLG-E	L-NMP	29500	Y	4	
133	KLG-E	L-NMP	29500	Y	4	
147	KLG-E	L-NMP	23032	Y	5	
152	KLG-E	L-NMP	17394	Y	4	
155	KLG-E	L-NMP	17394	Y	4	
103	KLG-E	L-EoMP	1738	N	NA	4
104	KLG-E	L-EoMP	1738	N	NA	
124	KLG-E	L-EoMP	40	N	NA	
125	KLG-E	L-EoMP	40	N	NA	
134	KLG-E	L-EoMP	91	Y	5	

<table>
<tr><td>135</td><td>KLG-E</td><td>L-EoMP</td><td>91</td><td>Y</td><td>4</td><td rowspan="2"></td></tr>
<tr><td>148</td><td>KLG-E</td><td>L-EoMP</td><td>288</td><td>N</td><td>NA</td></tr>
<tr><td>128</td><td>KLG-E</td><td>L-EB</td><td>1545</td><td>Y</td><td>16</td><td rowspan="6">11</td></tr>
<tr><td>129</td><td>KLG-E</td><td>L-EB</td><td>1545</td><td>Y</td><td>6</td></tr>
<tr><td>130</td><td>KLG-E</td><td>L-EB</td><td>122</td><td>N</td><td>NA</td></tr>
<tr><td>131</td><td>KLG-E</td><td>L-EB</td><td>122</td><td>N</td><td>NA</td></tr>
<tr><td>154</td><td>KLG-E</td><td>L-EB</td><td>260</td><td>N</td><td>NA</td></tr>
<tr><td>156</td><td>KLG-E</td><td>L-EB</td><td>260</td><td>N</td><td>NA</td></tr>
</table>

Bulk and purified populations used for secondary transplantation to identify leukemic propagating population. Number of cells used for transplanted, whether the mice developed a leukemia, and the latency if mice developed a leukemia are shown. L-NMP: leukemic neutrophil-monocyte progenitor; L-EoMP: leukemic eosinophil-mast cell progenitor; L-EB: leukemic erythroblast; Y, yes; N, No; NA, not applicable.

A

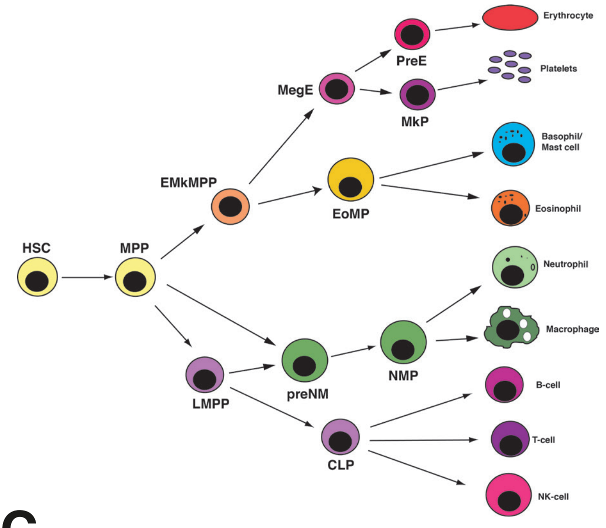

B

EMkMPP	Erythroid-megakaryocyte primed multi-potent progenitor	Lin–Sca1–c-Kit+CD41–FcγRII/III–CD150–CD105–CD55+	Drissen et al. 2016
EoMP	Eosinophil mast cell progenitor	Lin–Sca1–c-Kit+CD41–FcγRII/III+CD150–CD55+	Drissen et al. 2016
MegE	Megakaryocyte erythroid progenitor	Lin–Sca1–c-Kit+CD41–FcγRII/III–CD150+CD105–	Pronk et al. 2007
MkP	Megakaryocyte progenitor	Lin–Sca1–c-Kit+CD41+ CD150+	Pronk et al. 2007
preCFU-E	Pre-colony forming unit erythroid progenitor	Lin–Sca1–c-Kit+CD41–FcγRII/III–CD150+CD105+	Pronk et al. 2007
CFU-E	Colony forming unit erythroid progenitor	Lin–Sca1–c-Kit+CD41–FcγRII/III–CD150–CD105+	Pronk et al. 2007
LMPP	Lymphoid-primed multi-potent progenitor	Lin–Sca1+c-Kit+Flt3hi	Adolfsson et al. 2005
preNM	Pre-neutrophil-monocyte progenitor	Lin–Sca1–c-Kit+CD41–FcγRII/III–CD150–CD105–CD55–	Drissen et al. 2016
NMP	Neutrophil-monocyte progenitor	Lin–Sca1–c-Kit+CD41–FcγRII/III+CD150–CD55–	Drissen et al. 2016
Stage I EB	Stage I erythroblast/pro-erythroblast	CD71hiTer119lo	Socolovsky et al. 2001
Stage II EB	Stage II erythroblast/basophilic erythroblast	CD71hiTer119hi	Socolovsky et al. 2001
Stage III EB	Stage III erythroblast/late basophilic erythroblast	CD71intTer119hi	Socolovsky et al. 2001
Stage IV EB	Stage IV erythroblast/ orthochromatophilic erythroblast	CD71loTer119hi	Socolovsky et al. 2001

C

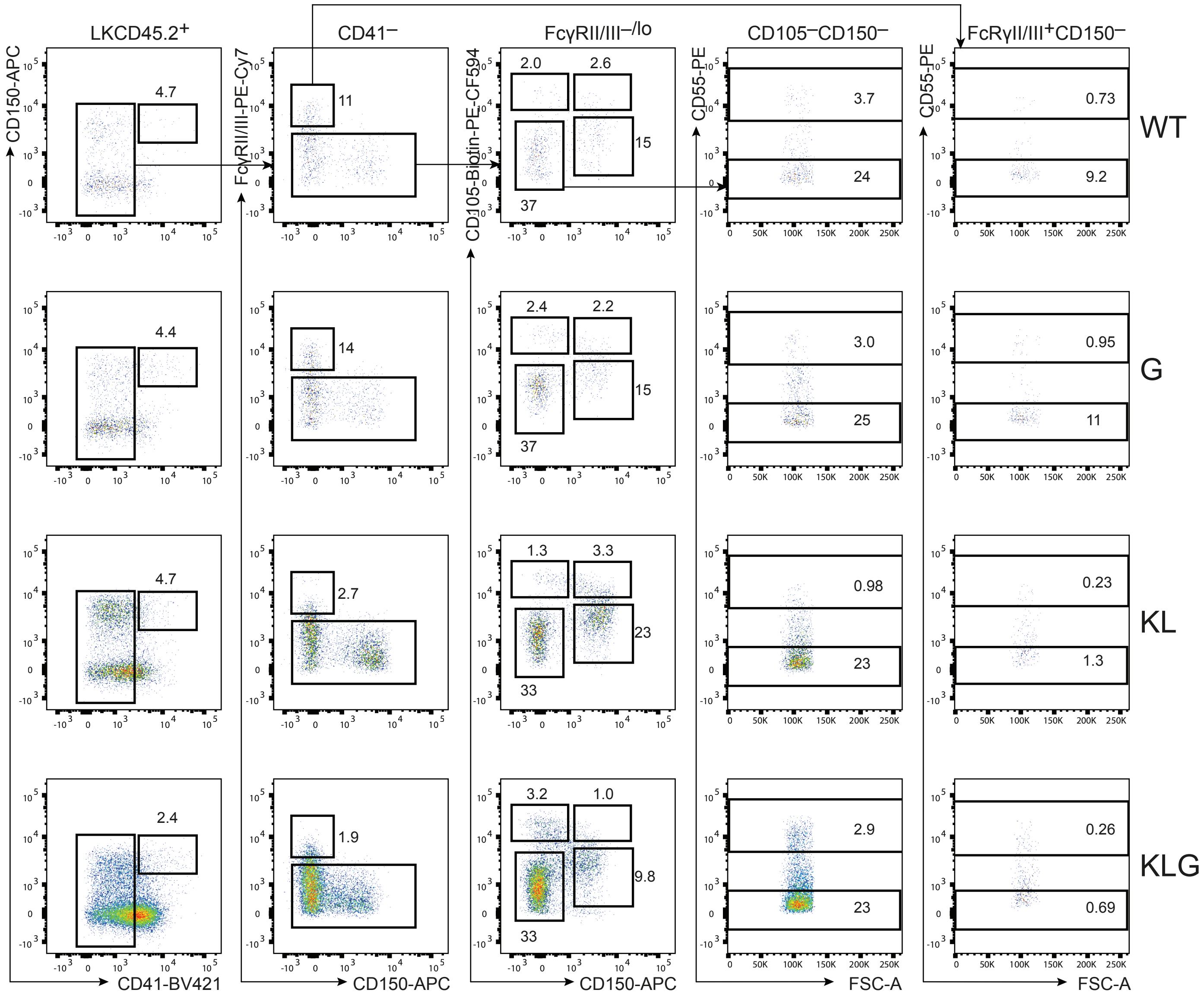

Figure S5

Figure S5, related to Figure 3. Analysis of the myelo-erythroid progenitors in pre-leukemic and leukemia mice.

(A) Schematic of the hematopoietic hierarchy.

(B) Table of surface markers used to distinguish each progenitor population.

(C) Representative flow cytometry plots of phenotyping of BM myelo-erythroid progenitor at 6 weeks post-fetal liver (FL) transplantation and their quantification as a percentage of $Lin^{-}c\text{-}Kit^{+}$ (LK) cells across all experiments.

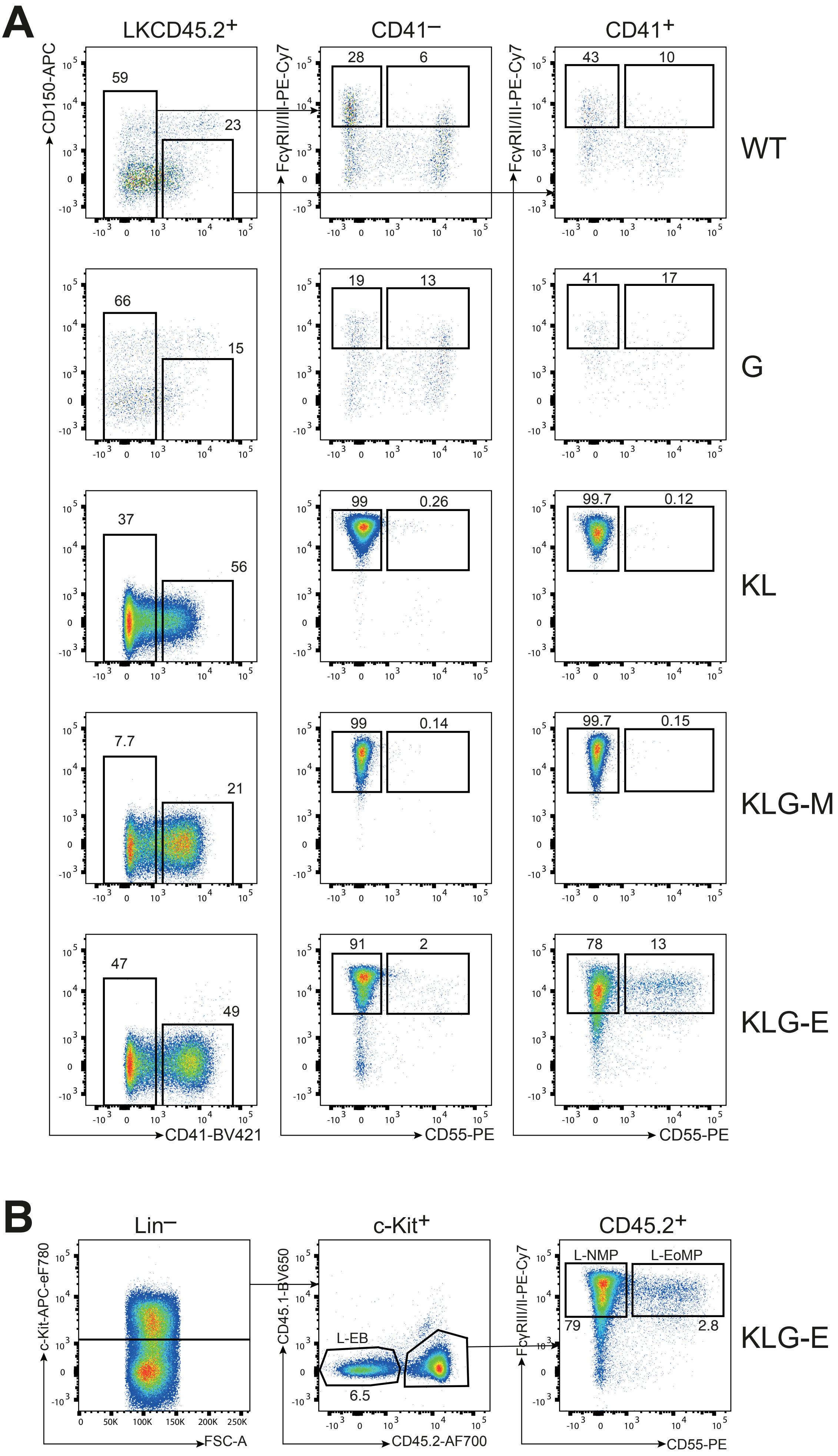
A
LKCD45.2+
CD41−
CD41+
CD150-APC
FcγRII/III-PE-Cy7
CD41-BV421
CD55-PE
WT
G
KL
KLG-M
KLG-E
59
23
28
6
43
10
66
15
19
13
41
17
37
56
99
0.26
99.7
0.12
7.7
21
99
0.14
99.7
0.15
47
49
91
2
78
13
B
Lin−
c-Kit+
CD45.2+
c-Kit-APC-eF780
FSC-A
CD45.1-BV650
CD45.2-AF700
FcγRIII/II-PE-Cy7
L-EB
6.5
L-NMP
L-EoMP
79
2.8

Figure S6

Figure S6, related to Figure 3. *Cebpa* and *Gata2* mutant AEL is sustained by leukemia-initiating cells (LICs) with a NMP immuno-phenotype.

(A) Representative flow cytometry analysis of experimental myelo-erythroid cells as a percentage of LKCD45.2^{+} cells in terminal analysis of BM from transplanted mice with the indicated genotypes and leukemia phenotypes. Note that both CD41^{+} and CD41^{-} cells from leukemic, but not from non-leukemic mice are predominantly FcγRII/III^{+}, and that FcγRII/III^{+}CD55^{+} leukemic cells are only observed in KLG-E mice. The number of gated cells as percentage of the parental gate is shown.

(B) Gating strategy for purifying L-NMP from KLG-M and KLG-E mice, and L-EoMP and L-EB from KLG-E mice for secondary transplantations. For L-EB, L-EoMP and L-NMP the number of cells as a percentage of the LK population is shown, n=5.

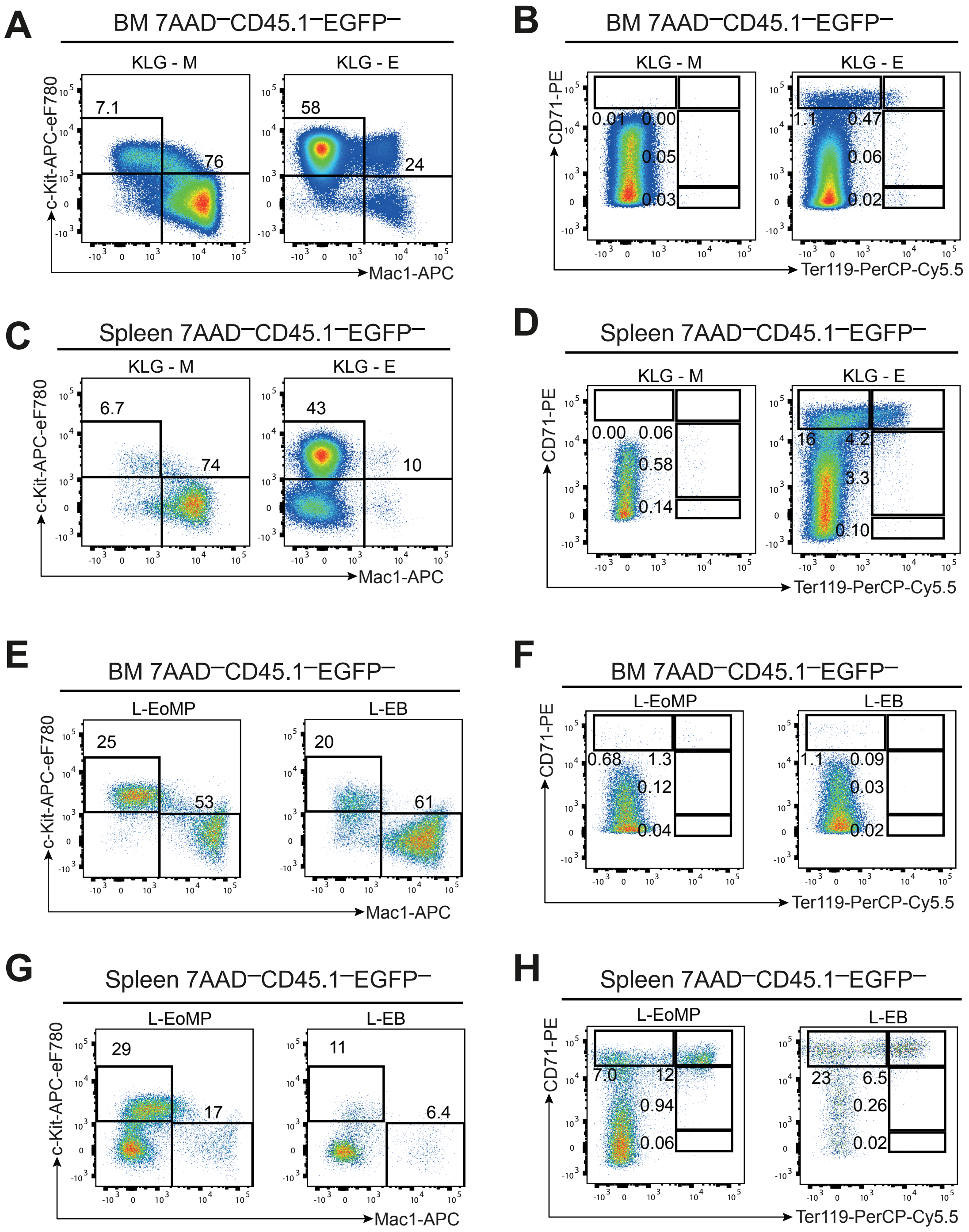
A
BM 7AAD⁻CD45.1⁻EGFP⁻
KLG - M
KLG - E
c-Kit-APC-eF780
Mac1-APC
7.1
76
58
24
B
BM 7AAD⁻CD45.1⁻EGFP⁻
KLG - M
KLG - E
CD71-PE
Ter119-PerCP-Cy5.5
0.01
0.00
0.05
0.03
1.1
0.47
0.06
0.02
C
Spleen 7AAD⁻CD45.1⁻EGFP⁻
KLG - M
KLG - E
c-Kit-APC-eF780
Mac1-APC
6.7
74
43
10
D
Spleen 7AAD⁻CD45.1⁻EGFP⁻
KLG - M
KLG - E
CD71-PE
Ter119-PerCP-Cy5.5
0.00
0.06
0.58
0.14
16
4.2
3.3
0.10
E
BM 7AAD⁻CD45.1⁻EGFP⁻
L-EoMP
L-EB
c-Kit-APC-eF780
Mac1-APC
25
53
20
61
F
BM 7AAD⁻CD45.1⁻EGFP⁻
L-EoMP
L-EB
CD71-PE
Ter119-PerCP-Cy5.5
0.68
1.3
0.12
0.04
1.1
0.09
0.03
0.02
G
Spleen 7AAD⁻CD45.1⁻EGFP⁻
L-EoMP
L-EB
c-Kit-APC-eF780
Mac1-APC
29
17
11
6.4
H
Spleen 7AAD⁻CD45.1⁻EGFP⁻
L-EoMP
L-EB
CD71-PE
Ter119-PerCP-Cy5.5
7.0
12
0.94
0.06
23
6.5
0.26
0.02

Figure S7

Figure S7, related to Figure 3. Transplantation of L-NMPs re-capitulates the phenotype of the original disease.

(A) Representative flow cytometry analysis of c-Kit^{lo}Mac1^{+} and c-Kit^{+}Mac1^{lo} cells as a percentage of 7AAD^{-}CD45.1^{-}EGFP^{-} cells in the BM from mice transplanted with purified L-NMPs BM cells from KLG-M and KLG-E leukemic mice as described in Figure 3D.

(B) Representative flow cytometry analysis of stage I-IV erythroblast cells as a percentage of 7AAD^{-} CD45.1^{-}EGFP^{-} cells in the BM from mice transplanted with purified L-NMPs BM cells from KLG-M and KLG-E leukemic mice as described in Figure 3E.

(C) Representative flow cytometry analysis of c-Kit^{lo}Mac1^{+} and c-Kit^{+}Mac1^{lo} cells as a percentage of 7AAD^{-}CD45.1^{-}EGFP^{-} cells in the spleen from mice transplanted with purified L-NMPs BM cells from KLG-M and KLG-E leukemic mice as described in Figure 3D.

(D) Representative flow cytometry analysis of stage I-IV erythroblast cells as a percentage of 7AAD^{-} CD45.1^{-}EGFP^{-} cells in the spleen from mice transplanted with purified L-NMPs BM cells from KLG-M and KLG-E leukemic mice as described in Figure 3E.

(E) Representative flow cytometry analysis of c-Kit^{lo}Mac1^{+} and c-Kit^{+}Mac1^{lo} cells as a percentage of 7AAD^{-}CD45.1^{-}EGFP^{-} cells in the BM from mice transplanted with purified L-EoMPs and L-EBs BM cells from KLG-E leukemic mice as described in Figure 3D.

(F) Representative flow cytometry analysis of stage I-IV erythroblast cells as a percentage of 7AAD^{-} CD45.1^{-}EGFP^{-} cells in the BM from mice transplanted with purified L-EoMPs and L-EBs BM cells from KLG-M and KLG-E leukemic mice as described in Figure 3E.

(G) Representative flow cytometry analysis of c-Kit^{lo}Mac1^{+} and c-Kit^{+}Mac1^{lo} cells as a percentage of 7AAD^{-}CD45.1^{-}EGFP^{-} cells in the spleen from mice transplanted with purified L-EoMPs and L-EBs BM cells from KLG-E leukemic mice as described in Figure 3D.

(H) Representative flow cytometry analysis of stage I-IV erythroblast cells as a percentage of 7AAD^{-} CD45.1^{-}EGFP^{-} cells in the spleen from mice transplanted with purified L-EoMPs and L-EBs BM cells from KLG-M and KLG-E leukemic mice as described in Figure 3E.

Table S3, Related to Figure 3. Limiting dilution of LIC populations.

Cell type	50 cells	200 cells	500 cells
L-NMP	1/4	2/4	2/3
L-EoMP	1/4	4/4	4/4
L-EB	0/4	0/4	0/4

For each of the cell type-cell number combinations the fraction of mice engrafted (defined as >1% leukemic BM cells) 8 weeks after transplantation is given.

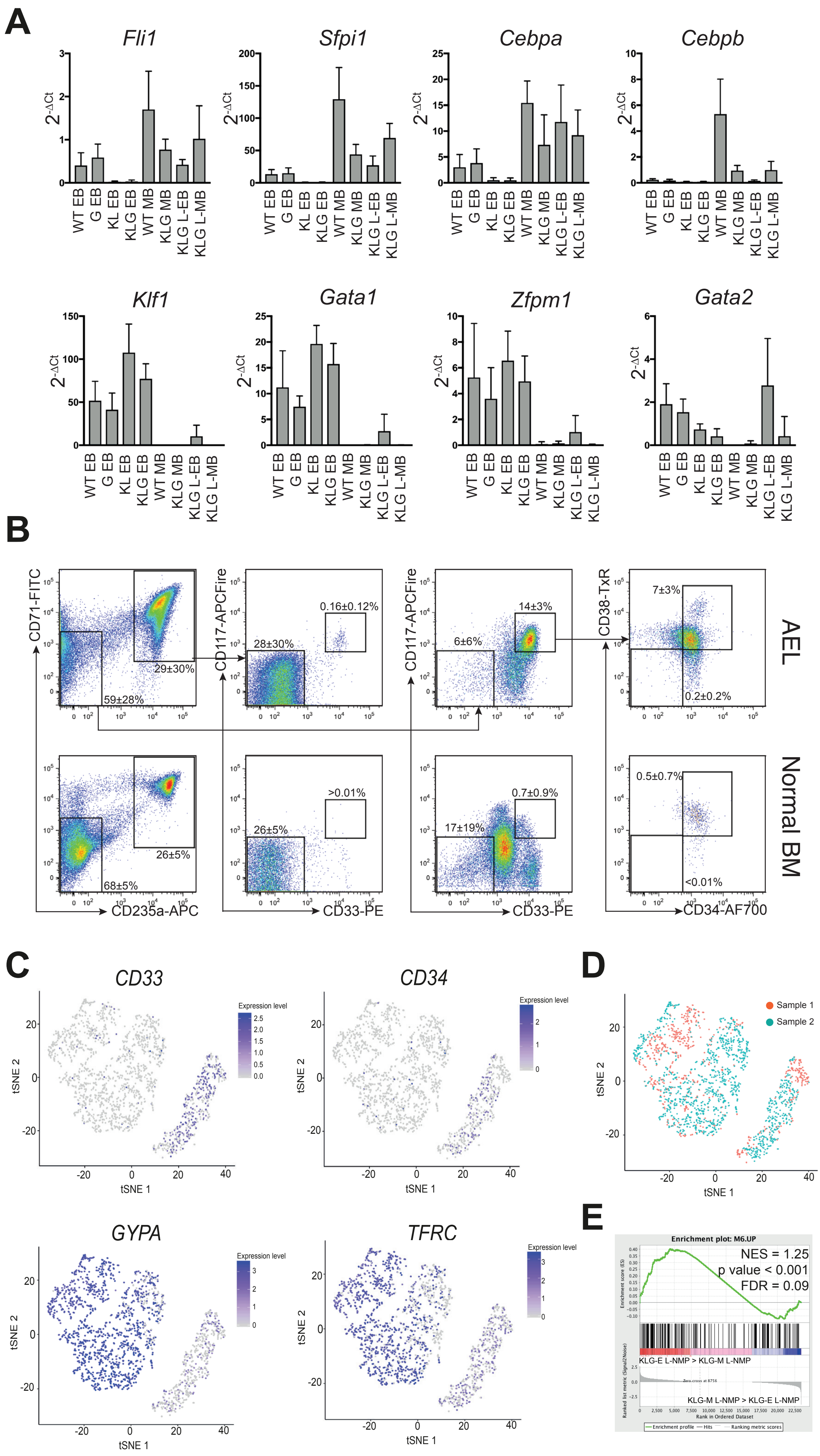
A
Fli1
Sfpi1
Cebpa
Cebpb
Klf1
Gata1
Zfpm1
Gata2
2-ΔCt
WT EB
G EB
KL EB
KLG EB
WT MB
KLG MB
KLG L-EB
KLG L-MB
B
CD71-FITC
CD117-APCFire
CD38-TxR
CD235a-APC
CD33-PE
CD34-AF700
AEL
Normal BM
29±30%
59±28%
0.16±0.12%
28±30%
14±3%
6±6%
7±3%
0.2±0.2%
26±5%
68±5%
>0.01%
26±5%
0.7±0.9%
17±19%
0.5±0.7%
<0.01%
C
CD33
CD34
GYPA
TFRC
Expression level
tSNE 1
tSNE 2
D
Sample 1
Sample 2
E
Enrichment plot: M6.UP
NES = 1.25
p value < 0.001
FDR = 0.09
KLG-E L-NMP > KLG-M L-NMP
KLG-M L-NMP > KLG-E L-NMP
Rank in Ordered Dataset
Enrichment profile
Hits
Ranking metric scores

Figure S8

Figure S8, related to Figure 4. Murine AEL model is analogous to human AEL.

(A) Multiplex qRT-PCR of myeloid and erythroid transcription factor (TF) genes on bulk 50 EBs and myeloblast (MBs) from WT, G, KL, KLG mice 6 weeks post-FL transplantation and KLG-E leukemic mice. Values shown are $2^{-\Delta Ct}$ normalized to *Hprt* ± SD averaged from 2-3 biological and 2-4 technical replicates per biological sample.

(B) Representative flow cytometry analysis of human AEL (upper panels; n=4) and normal BM (n=2). The gating strategy used to quantify $CD235a^{-}CD71^{lo}CD33^{+}CD34^{+}KIT^{+}$ LIC-like progenitors is shown, as is the average abundance (as % of live singlets) of the gated populations.

(C) tSNEs plot of human AEL single cells showing the expression of *CD33*, *CD34, GYPA* and *TFRC.* No minimum cut off was used.

(D) tSNE plot of human AEL single cells showing the sample to which individual cells belong.

(E) GSEA of KLG-E L-NMP vs. KLG-M L-NMP (n=3/genotype) using a M6 gene set. Normalized enrichment score (NES), p value and false discovery rate (FDR) are indicated.

Table S7. Related to STAR methods. Primers and probes

Gene/Genomic region	Sequence/Assay ID	Assay
Gata2^{G320D} Forward	GTAGCCCTCTCTTCCGTGTT	PCR
Gata2^{G320D} Reverse	GCCACAGTTGTCCAGGATTG	PCR
Cebpa^{Lp30} Forward	GCACCAGACGTCTATAGAC	PCR
Cebpa^{Lp30} Reverse	CTGTCGGCTGTGCTGGAAGA	PCR
Cebpa^{K313KK} Forward	CGCTGGTGATCAAACAAGAG	PCR
Cebpa^{K313KK} Reverse	CTCGTTGCTGTTCTTGTCCA	PCR
Barcode Forward (1)	TCGTCGGCAGCGTCAGATGTGTATAAGAGACAGT GGCATGGACGAGCTGTACCAG	PCR
Barcode Forward (2)	TCGTCGGCAGCGTCAGATGTGTATAAGAGACAGA TGGCATGGACGAGCTGTACCAG	PCR
Barcode Forward (3)	TCGTCGGCAGCGTCAGATGTGTATAAGAGACAGG ATGGCATGGACGAGCTGTACCAG	PCR
Barcode Reverse	GTCTCGTGGGCTCGGAGATGTGTATAAGAGACAG GAAAGCCATACGGGAAGCAATAGC	PCR
Ad1_noMX	AATGATACGGCGACCACCGAGATCTACACTCGTC GGCAGCGTCAGATGTG	PCR
Ad2.1_TAAGGCGA	CAAGCAGAAGACGGCATACGAGATTCGCCTTAGT CTCGTGGGCTCGGAGATGT	PCR
Ad2.2_CGTACTAG	CAAGCAGAAGACGGCATACGAGATCTAGTACGGT CTCGTGGGCTCGGAGATGT	PCR
Ad2.3_AGGCAGAA	CAAGCAGAAGACGGCATACGAGATTTCTGCCTGT CTCGTGGGCTCGGAGATGT	PCR
Ad2.4_TCCTGAGC	CAAGCAGAAGACGGCATACGAGATGCTCAGGAGT CTCGTGGGCTCGGAGATGT	PCR
Ad2.5_GGACTCCT	CAAGCAGAAGACGGCATACGAGATAGGAGTCCGT CTCGTGGGCTCGGAGATGT	PCR
Ad2.6_TAGGCATG	CAAGCAGAAGACGGCATACGAGATCATGCCTAGT CTCGTGGGCTCGGAGATGT	PCR
Ad2.7_CTCTCTAC	CAAGCAGAAGACGGCATACGAGATGTAGAGAGGT CTCGTGGGCTCGGAGATGT	PCR
Ad2.8_CAGAGAGG	CAAGCAGAAGACGGCATACGAGATCCTCTCTGGT CTCGTGGGCTCGGAGATGT	PCR
Ad2.9_GCTACGCT	CAAGCAGAAGACGGCATACGAGATAGCGTAGCGT CTCGTGGGCTCGGAGATGT	PCR

Ad2.10_CGAGGCTG	CAAGCAGAAGACGGCATACGAGATCAGCCTCGGT CTCGTGGGCTCGGAGATGT	PCR
Ad2.11_AAGAGGCA	CAAGCAGAAGACGGCATACGAGATTGCCTCTTGT CTCGTGGGCTCGGAGATGT	PCR
Ad2.12_GTAGAGGA	CAAGCAGAAGACGGCATACGAGATTCCTCTACGT CTCGTGGGCTCGGAGATGT	PCR
Ad2.13_GTCGTGAT	CAAGCAGAAGACGGCATACGAGATATCACGACGT CTCGTGGGCTCGGAGATGT	PCR
Ad2.14_ACCACTGT	CAAGCAGAAGACGGCATACGAGATACAGTGGTGT CTCGTGGGCTCGGAGATGT	PCR
Ad2.15_TGGATCTG	CAAGCAGAAGACGGCATACGAGATCAGATCCAGT CTCGTGGGCTCGGAGATGT	PCR
Ad2.16_CCGTTTGT	CAAGCAGAAGACGGCATACGAGATACAAACGGGT CTCGTGGGCTCGGAGATGT	PCR
Ad2.17_TGCTGGGT	CAAGCAGAAGACGGCATACGAGATACCCAGCAGT CTCGTGGGCTCGGAGATGT	PCR
Ad2.18_GAGGGGTT	CAAGCAGAAGACGGCATACGAGATAACCCCTCGT CTCGTGGGCTCGGAGATGT	PCR
Ad2.19_AGGTTGGG	CAAGCAGAAGACGGCATACGAGATCCCAACCTGT CTCGTGGGCTCGGAGATGT	PCR
Ad2.20_GTGTGGTG	CAAGCAGAAGACGGCATACGAGATCACCACACGT CTCGTGGGCTCGGAGATGT	PCR
Ad2.21_TGGGTTTC	CAAGCAGAAGACGGCATACGAGATGAAACCCAGT CTCGTGGGCTCGGAGATGT	PCR
Ad2.22_TGGTCACA	CAAGCAGAAGACGGCATACGAGATTGTGACCAGT CTCGTGGGCTCGGAGATGT	PCR
Ad2.23_TTGACCCT	CAAGCAGAAGACGGCATACGAGATAGGGTCAAGT CTCGTGGGCTCGGAGATGT	PCR
Ad2.24_CCACTCCT	CAAGCAGAAGACGGCATACGAGATAGGAGTGGGT CTCGTGGGCTCGGAGATGT	PCR
Gata2	Mm00492301_m1	TaqMan probe
Gata1	Mm01352636_m1	TaqMan probe
Zfpm1	Mm00494336_m1	TaqMan probe
Klf1	Mm00516096_m1	TaqMan probe
Gfi1	Mm00515855_m1	TaqMan probe
Irf8	Mm00492567_m1	TaqMan probe
Cebpb	Mm00843434_s1	TaqMan probe

Klf4	Mm00516104_m1	TaqMan probe
Cebpa	Mm00514283_s1	TaqMan probe
Sfpi1	Mm00488142_m1	TaqMan probe
Fli1	Mm00484410_m1	TaqMan probe
Ikzf2	Mm00496108_m1	TaqMan probe
Ikzf1	Mm01187882_m1	TaqMan probe
Etv6	Mm00468390_m1	TaqMan probe
Nfe2	Mm00801891_m1	TaqMan probe
Runx1	Mm01213404_m1	TaqMan probe
Cebpe	Mm02030363_s1	TaqMan probe
Ctsg	Mm00456011_m1	TaqMan probe
Elane	Mm00469310_m1	TaqMan probe
Mpo	Mm01298424_m1	TaqMan probe
Prtn3	Mm00478323_m1	TaqMan probe
Gfi1b	Mm00492318_m1	TaqMan probe
Gypa	Mm00494848_m1	TaqMan probe

Sequences of oligonucleotides used for genotyping and sequencing library preparation (PCR), and identity of probes used for qRT-PCR (TaqMan probes).

Table S8, Related to STAR methods. Mouse antibodies and viability dyes used for each staining panel

Antigen	Conjugate	Panel
7-Aminoactinomycin D (7AAD)	PE-Cy5	HSC, myelo-erythroid progenitor, erythroblast, WBC
CD4	APC-eF780	WBC
CD8a	APC-eF780	WBC
NK1.1	PB	WBC
Gr1	PO	WBC
CD19	PE-Cy7	WBC
Mac1	APC	Erythroblast, WBC
CD45.1	PE	WBC
CD45.2	AF700	HSC, myelo-erythroid progenitor, erythroblast, WBC
CD4	PE-Cy5	HSC, myelo-erythroid progenitor
CD8a	PE-Cy5	HSC, myelo-erythroid progenitor
Ter119	PE-Cy5	HSC, myelo-erythroid progenitor
Mac1	PE-Cy5	HSC, myelo-erythroid progenitor
Gr1	PE-Cy5	HSC, myelo-erythroid progenitor
CD150	APC	Myelo-erythroid progenitor
c-Kit	APC-eF780	HSC, myelo-erythroid progenitor, erythroblast
CD45.1	BV650	HSC, myelo-erythroid progenitor, erythroblast
CD48	APC	HSC
CD150	PE-Cy7	HSC, erythroblast, platelet and erythrocytes
Sca-1	PB	HSC
Streptavidin	PE-TxRed	Myelo-erythroid progenitor
Flt3	PE	HSC
CD5	PE-Cy5	HSC, myelo-erythroid progenitor
B220	PE-Cy5	HSC, myelo-erythroid progenitor
FcγRII/III	PE-Cy7	Myelo-erythroid progenitor
Sca1	BV605	Myelo-erythroid progenitor
CD105	Biotin	Myelo-erythroid progenitor
CD41	BV421	Myelo-erythroid progenitor, erythroblast
CD55	PE	Myelo-erythroid progenitor
CD71	PE	Myelo-erythroid progenitor
Ter119	PerCP-Cy5.5	Erythroblast, platelet and erythrocytes
CD41	PE	Platelet and erythrocytes

Antibodies used for flow cytometry and cell sorting. The antigen, fluorophore and staining panels in which the antibody was used are shown.
